# Photochemically Induced Cyclometalations at Simple Platinum(II) Precursors

**DOI:** 10.1021/acs.inorgchem.3c00688

**Published:** 2023-04-12

**Authors:** Dionisio Poveda, Ángela Vivancos, Delia Bautista, Pablo González-Herrero

**Affiliations:** † Departamento de Química Inorgánica, Facultad de Química, Universidad de Murcia, Campus de Espinardo, 19, 30100 Murcia, Spain; ‡ Área Científica y Técnica de Investigación, Universidad de Murcia, Campus de Espinardo, 21, 30100 Murcia, Spain

## Abstract

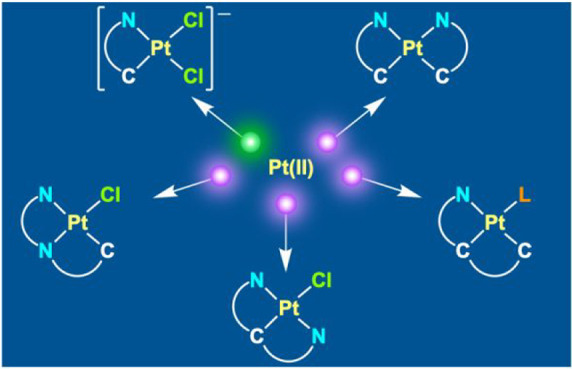

Photochemical cycloplatinations of 2-arylpyridines and related C∧N ligands, as well as terdentate heteroaromatic N∧N∧C, N∧C∧N, and N∧C∧C compounds, are demonstrated using (Bu_4_N)_2_[Pt_2_Cl_6_] or [PtCl_2_(NCPh)_2_] as precursors at room temperature. Mono- or bis-cyclometalated Pt(II) complexes with C∧N ligands are obtained depending on excitation wavelength and precursor. Monitoring experiments show that photoexcitation enables both the N-coordination and the subsequent C–H metalation. Photochemical synthetic protocols have been developed, which are advantageous with respect to the established thermal procedures and have allowed the synthesis of the first Pt(II) complexes with N∧C∧C ligands.

## Abstract 2

Labilization of metal−ligand bonds through light irradiation at Pt(II) precursors promotes facile cyclometalation of heteroaromatic C∧N, N∧N∧C, N∧C∧N, and N∧C∧C compounds at room temperature. Advantageous photochemical protocols for the synthesis of cyclometalated Pt(II) complexes are demonstrated.

Cyclometalation is a fundamental organometallic reaction that has been widely exploited for the activation and functionalization of C–H bonds under thermal conditions^1−4^ and the synthesis of luminescent and photofunctional materials.^5−10^ Traditionally, photochemistry has been applied to the intermolecular activation of hydrocarbons by metal complexes, commonly to generate vacant coordination sites by photolysis using UV light.^11−17^ In limited cases, photolysis methods have led to the activation of C–H bonds of pendant alkyl or aryl groups of coordinated ligands, resulting in cyclometalated complexes.^12,18−25^ During the last two decades, photochemical strategies involving C–H activation have gained momentum, particularly within the fields of photoredox catalysis^26−28^ and light-induced transition metal catalysis,^29,30^ motivated by sustainability criteria. However, the development of light-based C–H activation methods for the synthesis of cyclometalated complexes has not been systematically addressed, and very few instances of photochemical cyclometalations have been reported in recent years.^31,32^ We and others have demonstrated the cyclometalation of 2-arylpyridines (N∧CH) through a photooxidative C–H addition that takes place upon visible-light irradiation of complexes [PtX(C∧N)(N∧CH)], resulting in bis-cyclometalated Pt(IV) hydrides (Scheme 1).^33−35^ This mechanism has been postulated as a key step in the C–H borylation of 2-arylpyridines catalyzed by Rh(I).^36^

**Scheme 1:**
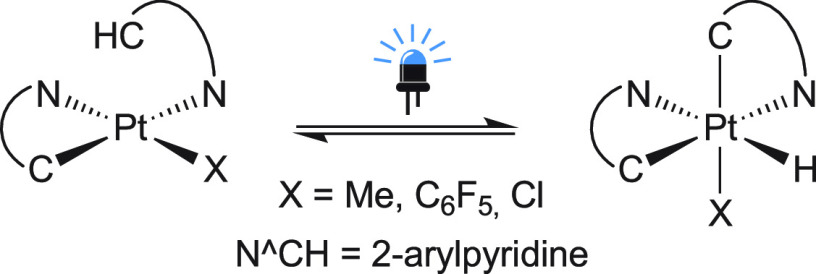
Photooxidative C–H Addition

Pt(II) complexes with cyclometalated 2-arylpyridines and related terdentate and tetradentate ligands constitute one of the most intensively studied classes of organometallic compounds because of their useful photophysical and photochemical properties, which make them suitable for diverse light-based applications,^37,38^ e.g., as phosphorescent dopants for organic light-emitting devices (OLEDs),^5,39−42^ probes for bioimaging,^43−45^ chemosensors,^46−49^ or photoredox catalysts.^50−53^ The development of new cyclometalated Pt(II) complexes critically depends on the availability of convenient synthetic methodologies. The cycloplatination of 2-arylpyridines and related C∧N ligands is commonly achieved under thermal conditions, most often by heating K_2_[PtCl_4_] and the ligand in alcohol/water mixtures.^54−60^ This method often presents disadvantages, such as partial decomposition to Pt(0). To avoid this, cycloplatinations using (Bu_4_N)_2_[PtCl_4_] have been performed at lower temperatures in alcohols, which requires reactions times of up to a week.^61−63^ More stable Pt(II) precursors have also been employed, such as *cis*-[PtCl_2_(DMSO)_2_].^64^ Alternatively, organometallic precursors like [Pt_2_Me_4_(μ-SMe_2_)_2_]^65,66^ and [Pt_2_(μ-Cl)_2_(η^3^-allyl)_2_]^67^ have been utilized to cycloplatinate C∧N ligands under mild conditions. Pt(II) complexes with terdentate N∧N∧C,^68−71^ N∧C∧N^72−75^ or C∧N∧C^76,77^ heteroaromatic ligands are usually synthesized thermally from K_2_[PtCl_4_] in MeCN/water or AcOH; the introduction of N∧C∧N and C∧N∧C ligands is particularly difficult and requires heating times of 3 days. Microwave-assisted cycloplatinations of C∧N^78^ and N∧C∧N^75^ ligands have also been developed, which require a strict control of irradiation power, time, and temperature.

Herein, we explore the photoreactivity of (Bu_4_N)_2_[Pt_2_Cl_6_] and [PtCl_2_(NCPh)_2_] in the presence of potentially bi- or terdentate heteroaromatic compounds with the aim to develop cycloplatination protocols under mild photochemical conditions. We initially tested the reaction of (Bu_4_N)_2_[Pt_2_Cl_6_] with 2-phenylpyridine (ppyH) and a base under irradiation with green light (λ_max_ = 516 nm) in acetone at room temperature, which gave Bu_4_N[PtCl_2_(ppy)] (**1a**; Scheme 2). The optimal conditions were 1.2 equiv of ppyH, 2 equiv of the polymeric base (piperidinomethyl)polystyrene per Pt, and an irradiation time of 48 h, which afforded **1a** in 66% isolated yield. Higher molar proportions of ppyH produced small amounts of *trans*-*N,N*-[PtCl(ppy)(ppyH)] because of the reaction of **1a** with excess ppyH,^79^ whereas the use of inorganic bases like NaHCO_3_ or Na_2_CO_3_ produced some decomposition to Pt(0) and small amounts of unidentified side products that hindered purification. Other 2-arylpyridines afforded the corresponding Bu_4_N[PtCl_2_(C∧N)] complexes (**1b**–**f**) under the optimized conditions in 59–69% yields. 2-Phenylquinoline, benzoquinoline, and 2-benzoylpyridine were also successfully cycloplatinated, although the respective Bu_4_N[PtCl_2_(C∧N)] compounds could not be precipitated and were, therefore, derivatized to [Pt(acac)(C∧N)] (**2g**–**h**). In the cases of **2g** and **2h**, irradiations were prolonged for 72 h, but the yields were still particularly low because of the diminished coordination ability of the ligands as a consequence of increased steric hindrance or rigidity.

**Scheme 2:**
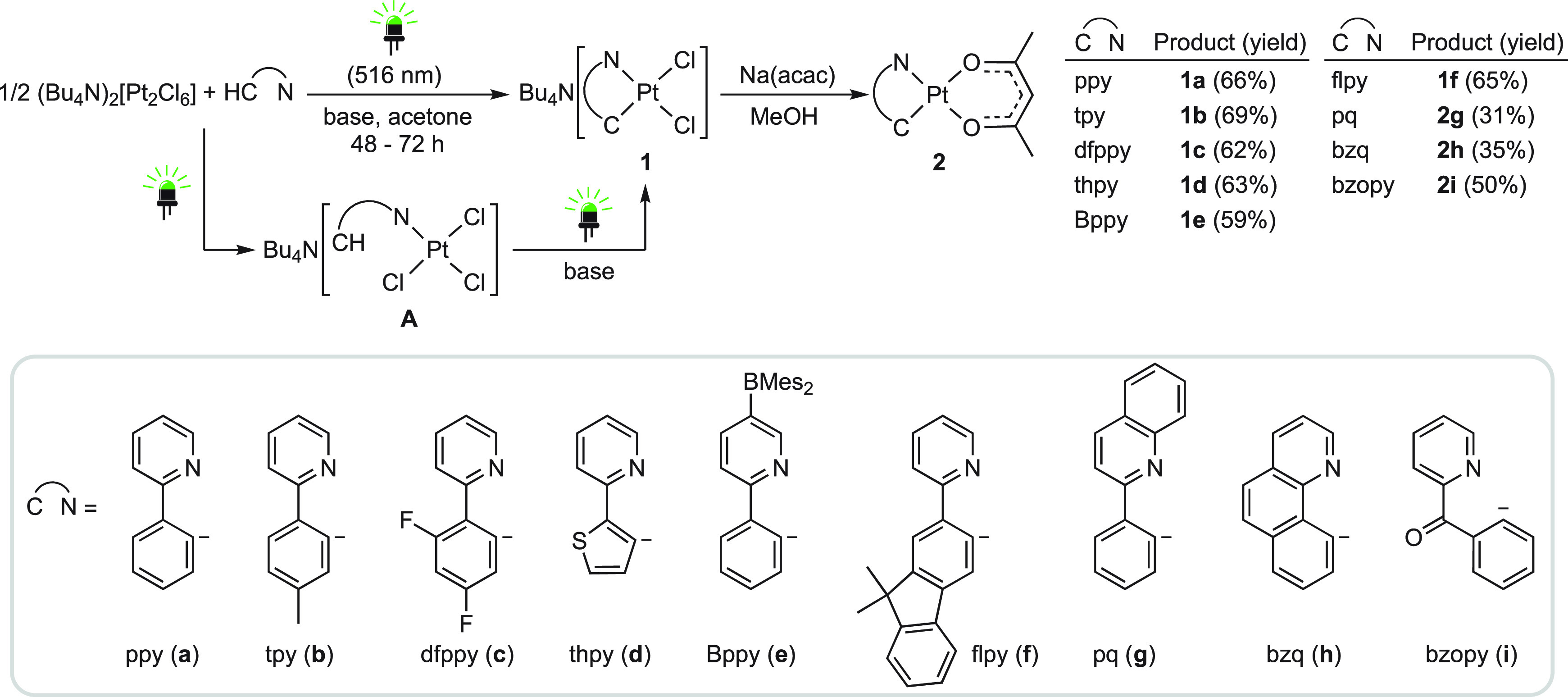
Photochemical Cycloplatinations Using (Bu_4_N)_2_[Pt_2_Cl_6_] and Proposed Pathway The base is (piperidinomethyl)polystyrene. Reaction times were 48 h for ligands **a–f**,**i** and 72 h for **g**,**h**.

Mechanistic insight into the observed cyclometalations was gained through several experiments. (Bu_4_N)_2_[Pt_2_Cl_6_] is stable in acetone, and no reaction with ppyH or 2-(*p*-tolyl)pyridine (tpyH) (1:2.2 molar ratio) was observed after 48 h at room temperature in the dark in the presence of (piperidinomethyl)polystyrene, thereby confirming that these cyclometalations require photoexcitation. The electronic absorption spectrum of (Bu_4_N)_2_[Pt_2_Cl_6_] in acetone (Figure 1a) displays two bands centered at 402 and 510 nm assignable to d–d transitions,^80^ which have dissociative character because of the involved dσ* orbital. The photoreactivity of this precursor was checked by registering its absorption spectrum in acetone after different times of irradiation with green light, which showed the gradual disappearance of the 510 nm band (Figure 1b) attributable to a photoinduced bridge-splitting to give Bu_4_N[PtCl_3_(acetone)]. A similar change in the absorption spectrum is observed upon titration of [(Me_2_N)_3_C_3_][Pt_2_Cl_6_] with olefins to give Zeise-type salts.^81^ It is then reasonable that irradiation with green light facilitates the coordination of 2-arylpyridines through the N atom, which would then undergo cyclometalation.

**Figure 1:**
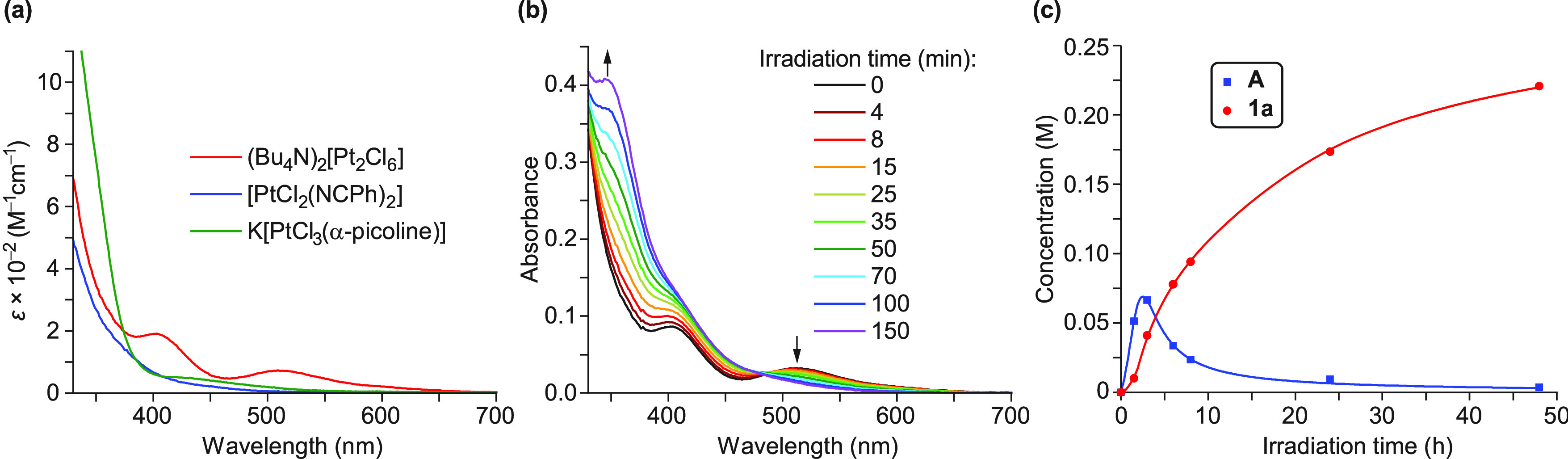
(a) Electronic absorption spectra of the Pt(II) precursors and K[PtCl_3_(α-picoline)] in acetone. (b) Absorption spectra of (Bu_4_N)_2_[Pt_2_Cl_6_] in acetone (ca. 5 × 10^–4^ M) after different times of irradiation with green light. (c) Plots of concentrations of **A** and **1a** vs irradiation time of an acetone-*d*_6_ solution of (Bu_4_N)_2_[Pt_2_Cl_6_] (0.018 M) and ppyH (0.040 M) with green light in the presence of piperidinomethyl(polystyrene).

Monitoring of an acetone-*d*_6_ solution of (Bu_4_N)_2_[Pt_2_Cl_6_], ppyH, and (piperidinomethyl)polystyrene (1:2:4) by ^1^H NMR after different times of irradiation with green LEDs (Figure S18) showed, indeed, the formation of an intermediate complex (**A**), whose concentration reaches a maximum after 3 h and then decreases as the concentration of **1a** gradually increases (Figure 1c). Although not isolable, intermediate **A** can be safely identified as [PtCl_3_(ppyH)]^−^ because its resonances demonstrate the presence of an N-coordinated ppyH. Complexes of the type [PtCl_3_(L)]^−^, where L is pyridine or a derivative thereof, have been reported and are moderately stable in solution;^82,83^ the most closely related to **A** bears an N^1^-coordinated 2,4′-bipyridine and undergoes metalation of the 4′-pyridyl ring in hot water.^83^ A second experiment showed that, after an initial irradiation period of 1 h, the concentration of **A** remained constant for 48 h in the dark and only upon resuming irradiation was it consumed to produce **1a** (Figure S19). Therefore, both the coordination of ppyH to give **A** and the subsequent transformation into **1a** are light-induced. Since an absorption spectrum of **A** could not be obtained, we registered the absorption spectrum of the related compound K[PtCl_3_(α-picoline)],^82^ which shows a weak band in the range 400–550 nm, assignable to a d–d transition (Figure 1a). It is likely that **A** absorbs in the same region, and therefore, irradiation with green light could promote chloride dissociation and trigger the electrophilic metalation of the ppyH ligand.

When acetone solutions of (Bu_4_N)_2_[Pt_2_Cl_6_] and an excess of ppyH were irradiated with a violet LED (λ_max_ = 405 nm) in the presence of a base, a mixture of **1a** and the bis-cyclometalated complex *cis*-[Pt(ppy)_2_] (**3a**) (ca. 2:1) was obtained. Therefore, the violet light appears to promote the coordination of a second ppyH ligand and its subsequent metalation but to an insufficient extent to constitute a good synthetic method for **3a**. The precursor [PtCl_2_(NCPh)_2_] was then tested, in the expectation that the coordination of the second ppyH would be easier. Consistent with this, the irradiation of an acetone solution of [PtCl_2_(NCPh)_2_] and ppyH (ca. 1:3) with violet light for 24 h in the presence of Na_2_CO_3_ produced **3a** as the major product (Scheme 3). Other 2-arylpyridines or 2-phenylquinoline gave the analogous complexes **3b**–**g** in 24–57% yields. Irradiation times were prolonged to 48 h for 2-(2-thienyl)pyridine and 2-phenylquinoline, but the latter still gave a low yield, which was attributed to its low coordinating ability.

**Scheme 3:**
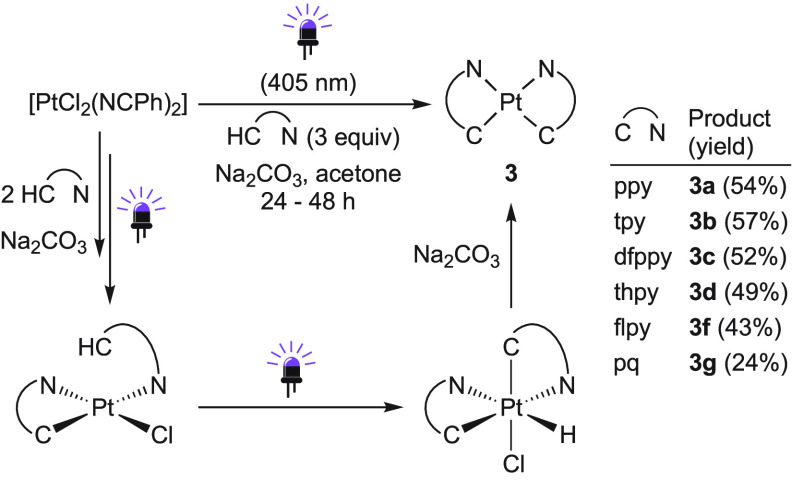
Photochemical Synthesis of *cis*-[Pt(C∧N)_2_] Complexes and Proposed Pathway Reaction times were 24 h for ligands **a–c**,**f** and 48 h for ligands **d**,**g**.

No reactions between [PtCl_2_(NCPh)_2_] and 2-arylpyridines were observed in acetone after 48 h in the dark in the presence of Na_2_CO_3_, which means that the described cyclometalations using this precursor are light-induced. The absorption spectrum of [PtCl_2_(NCPh)_2_] in acetone shows a tail in the range 350–500 nm assignable to d–d transitions (Figure 1a). Hence, this precursor absorbs very little in the green region and, in fact, it did not react with ppyH upon irradiation with green LEDs. In an attempt to detect intermediates, a solution of [PtCl_2_(NCPh)_2_] and tpyH (1:3) in acetone-*d*_6_ was irradiated with violet light in the absence of a base. After 5 min, the ^1^H NMR spectrum showed the Pt(IV) hydride [PtH(Cl)(tpy)_2_],^35^ whereas the only detected monocyclometalated intermediates were *cis*/*trans*-*N,N*-[PtCl(tpy)(tpyH)]^35,60^ (Figure S20). The data also revealed the presence of 2-(*p*-tolyl)pyridinium chloride, which resulted from the neutralization of HCl by tpyH. On the basis of these observations, we postulate that the first cyclometalation occurs through an electrophilic mechanism, although it is unclear if one or two 2-arylpyridines are coordinated before this step. In any case, the produced *cis*/*trans*-*N,N*-[PtCl(C∧N)(N∧CH)] isomers are known to interconvert photochemically, and the cis isomer produces [PtH(Cl)(C∧N)_2_] via C–H photooxidative addition;^35^ deprotonation by the base then leads to the *cis*-[Pt(C∧N)_2_] product (Scheme 3).

We next tested a series of potentially terdentate ligands (Scheme 4). The reactions between either of the Pt(II) precursors and 6-phenyl-2,2′-bipyridine (pbpyH) gave the desired complex [PtCl(pbpy)] (**4**) after 16 h of irradiation with violet light in acetone in the presence of a base. The highest yield was obtained using (Bu_4_N)_2_[Pt_2_Cl_6_], thereby representing a very convenient synthetic methodology. The attempts to cycloplatinate 1,3-di(2-pyridyl)benzene (dpybH) under the same conditions gave a mixture of unidentified species with a very low solubility (see the Supporting Information). We attribute this result to metalation at the C4 and/or C6 positions of the benzene ring, which has been previously shown to be kinetically favored over the more sterically hindered C2 position for Ir(III)^84^ and Pd(II).^72^ We therefore blocked the C4/C6 positions by using 4,6-difluoro-1,3-di(2-pyridyl)benzene (dfdpybH), which cleanly gave [PtCl(dfdpyb)] (**5**) in very good yields. In contrast, the dimetalation of 2,6-diphenylpyridine (dppyH) failed, reasonably because the steric hindrance of the phenyl rings makes the coordination step difficult.

**Scheme 4:**
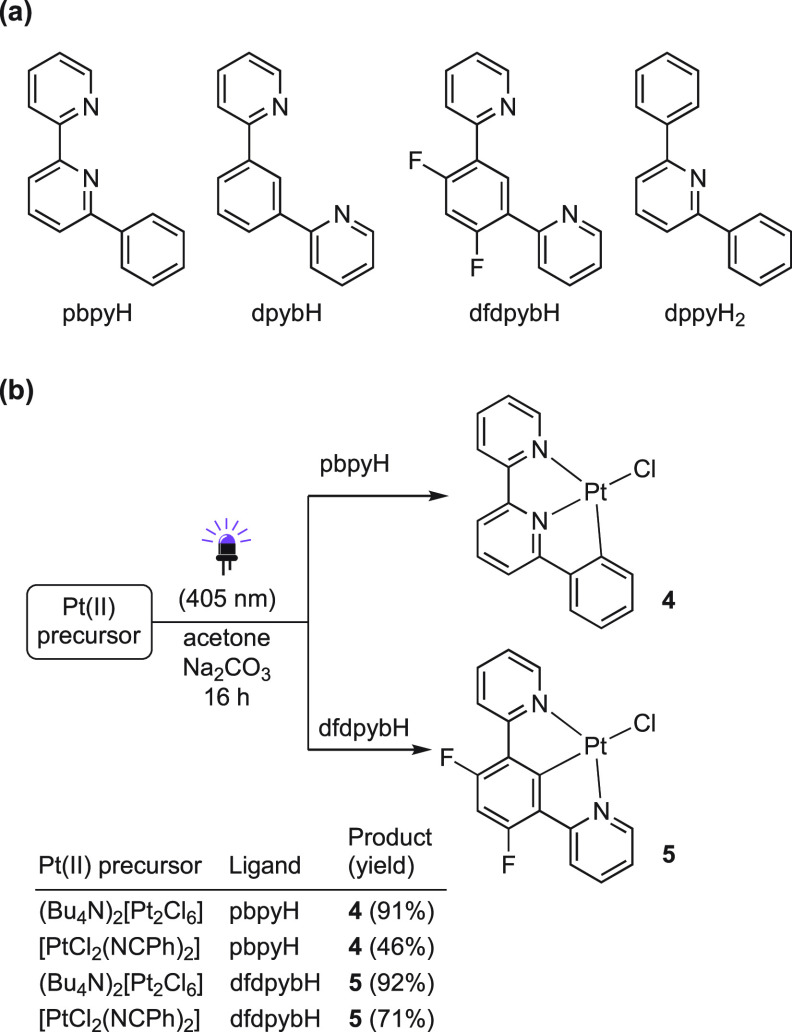
(a) Tested N∧N∧C, N∧C∧N, and C∧N∧C ligands for Photochemical Cyclometalations and (b) Successful Syntheses

The above results suggest that a relatively unhindered pyridyl ring is required to attain cyclometalation under mild photochemical conditions and that, if different positions are available for C–H metalation, the less hindered one will be favored. On the basis of these principles, we envisioned that 3,5-disubstituted 2-phenylpyridines like 2-(4,4″-dimethyl-[1,1′:3′,1″-terphenyl]-5′-yl)pyridine (dmtppyH_2_) and 2-(3,5-diphenoxyphenyl)pyridine (dPhOppyH_2_)^85^ (Scheme 5) would be suited for photochemical cyclometalation to attain a terdentate N∧C∧C coordination because the possible metalation positions are equivalent. Similar ligand designs have been employed to synthesize Au(III) N∧C∧C complexes, which require a microwave-assisted double C–H metalation from an N-coordinated precursor at high temperatures.^86,87^ The introduction of N∧C∧C ligands has also been achieved through the oxidative addition of 1-(2-pyridyl)-biphenylene to Ir(I),^88^ transmetalation to Au(III) from a 2-pyridyl-substituted dibenzostannol,^89^ or oxidative addition of 2-(2-pyridyl)-2,2′-diiodobiphenyl to Pd(0).^90^ However, there are no reports on the synthesis of Pt(II) complexes with heteroaromatic N∧C∧C ligands.

**Scheme 5:**
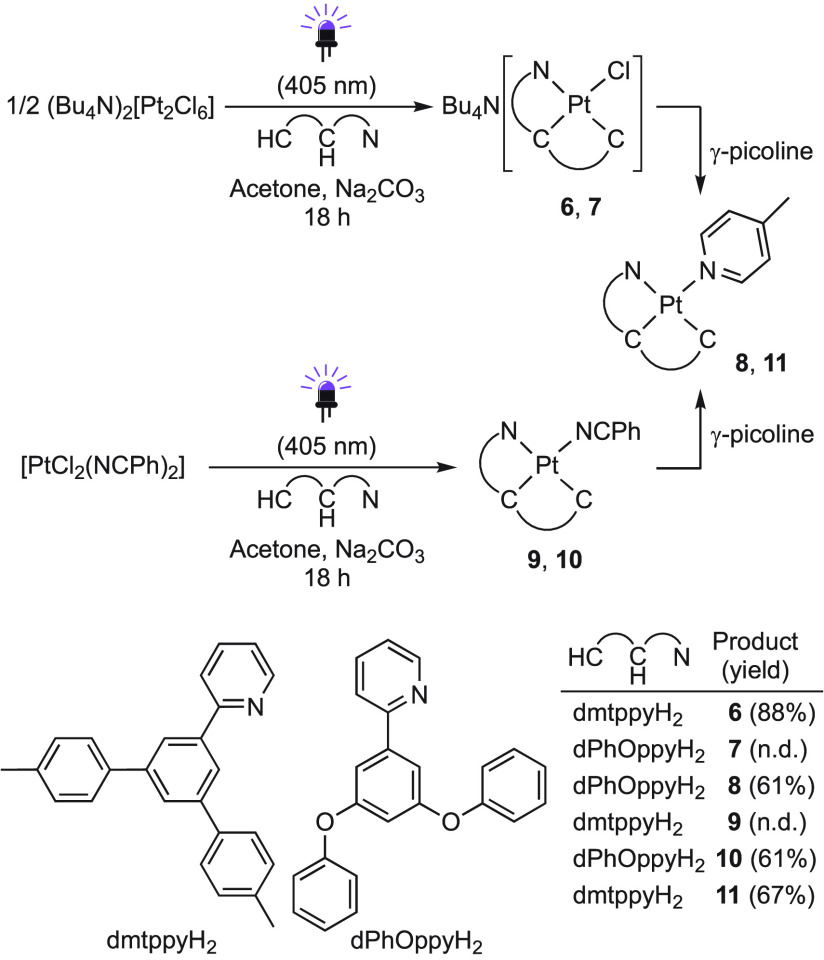
Photochemical Synthesis of Pt(II) N∧C∧C Complexes

In accordance with our expectations, dmtppyH_2_ and dPhOppyH_2_ underwent double C–H metalation to give Pt(II) N∧C∧C complexes under irradiation with violet light using either of the Pt(II) precursors and a base (Scheme 5). Starting from (Bu_4_N)_2_[Pt_2_Cl_6_], the compounds Bu_4_N[PtCl(N∧C∧C)] [N∧C∧C = dmtppy (**6**), dPhOppy (**7**)] were produced. Whereas **6** was isolated in good yield, **7** was found to be relatively unstable because of the lability of the chlorido ligand and was derivatized to [Pt(dPhOppy)(γ-picoline)] (**8**). The precursor [PtCl_2_(NCPh)_2_] led to the neutral complexes [Pt(N∧C∧C)(NCPh)] [N∧C∧C = dmtppy (**9**), dPhOppy (**10**)], of which **10** could be isolated in pure form, while isolation of **9** was unsuccessful, and therefore, it was converted into [Pt(dmtppy)(γ-picoline)] (**11**). The crystal structures of **8** and **11** (Figures S3 and S4) corroborate the N∧C∧C coordination of the dmtppy and dPhOppy ligands, respectively. We postulate that the two metalations leading to Pt(II) N∧C∧C complexes occur through consecutive electrophilic C–H activation steps that are facilitated by light-induced dissociation of chlorido or benzonitrile ligands. For comparison, thermal procedures were also attempted, which resulted in low yields or oxidation to Pt(IV) (Supporting Information).

In brief, the cyclometalation of heteroaromatic C∧N, N∧N∧C, N∧C∧N, and N∧C∧C ligands at Pt(II) can be induced photochemically under very mild conditions. With bidentate C∧N ligands, the outcome can be controlled through the choice of Pt(II) precursor and excitation wavelength, and NMR monitoring demonstrates that the coordination and metalation steps are light-induced. The photochemical procedures are advantageous relative to the established thermal methods in terms of energetic requirements and simplicity and have allowed the synthesis of the first Pt(II) complexes with N∧C∧C ligands. Derivatization and photophysical studies on [Pt(N∧C∧C)(L)] complexes are underway.

## Supporting Information Available

Experimental details and characterization data (PDF)
