# Supplementary material for: Photochemically Induced Cyclometalations at Simple Platinum(II) Precursors
Source: Inorg Chem. 2023 Apr 12;62(16):6207–13. doi: 10.1021/acs.inorgchem.3c00688 (PMC10131227; doi:10.1021/acs.inorgchem.3c00688)
Supplement: Supplementary file 1 — ic3c00688_si_001.pdf [file ic3c00688_si_001.pdf]

**SUPPORTING INFORMATION**

# Photochemically Induced Cyclometalations at Simple Platinum(II) Precursors

*Dionisio Poveda,[†] Ángela Vivancos,[†] Delia Bautista[‡] and Pablo González-Herrero[*,†]*

[†]Departamento de Química Inorgánica, Facultad de Química, Universidad de Murcia, Campus de Espinardo, 19, 30100 Murcia, Spain.

[‡]Área Científica y Técnica de Investigación, Universidad de Murcia, Campus de Espinardo, 21, 30100 Murcia, Spain.

*Email: pgh@um.es.

**Contents:**

1. Experimental details and characterization data ........ 2
   1.1. General considerations and materials ........ 2
   1.2. Spectroscopic and analytical methods ........ 2
   1.3. Synthesis of $(Bu_4N)_2[Pt_2Cl_6]$ ........ 3
   1.4. Synthesis of 2-(4,4''-dimethyl-[1,1':3',1''-terphenyl]-5'-yl)pyridine ($dmtppyH_2$) ........ 3
   1.5. Photochemical synthesis of monocyclometalated complexes $Bu_4N[PtCl_2(C^\wedge N)]$ (1) and $[Pt(C^\wedge N)(acac)]$ (2) ........ 3
   1.6. Photochemical synthesis of bis-cyclometalated complexes *cis*-$[Pt(C^\wedge N)_2]$ (3) ........ 5
   1.7. Photochemical synthesis of Pt(II) complexes with terdentade ligands ........ 5
   1.8. Reaction between $[PtCl_2(NCPh)_2]$ and 1,3-di(2-pyridyl)benzene (dpybH) under photochemical conditions ........ 8
   1.9. Thermal cycloplatinations of $dmtppyH_2$ and $dPhOppyH_2$ ........ 8
   1.10. X-Ray structure determinations ........ 10
2. NMR spectra of new compounds ........ 13
3. Reaction monitoring by $^1H$ NMR ........ 26
   3.1. Photochemical generation of $Bu_4N[PtCl_2(ppy)]$ from $(Bu_4N)_2[Pt_2Cl_6]$ and ppyH ........ 26
   3.2. Stability of $[PtCl_3(ppyH)]^-$ in the dark ........ 27
   3.3. Photochemical reaction between $[PtCl_2(NCPh)_2]$ and tpyH ........ 28
4. References ........ 29

# 1. Experimental details and characterization data

## 1.1. General considerations and materials

Photochemical reactions and NMR scale experiments were carried out at room temperature under an inert atmosphere. Synthesis-grade solvents were obtained from commercial sources. Acetone was thoroughly deoxygenated by bubbling nitrogen (syntheses) or performing three freeze-pump-thaw cycles (NMR monitoring). The compounds [Pd(PPh$_3$)$_4$],[1] [PtCl$_2$(NCPh)$_2$] (mixture of *cis* and *trans* isomers),[2] K[PtCl$_3$(α-picoline)],[3] Na(acac),[4] 5-(dimesitylboranyl)-2-phenylpyridine (BppyH),[5] 2-(9,9-dimethyl-9*H*-fluoren-2-yl)pyridine (flpyH),[6] 6-phenyl-2,2'-bipyridine (pbpyH),[7] 1,3-di(2-pyridyl)benzene (dpybH),[8] 4,6-difluoro-1,3-di(2-pyridyl)benzene (dfdpybH),[9] and 2-(3,5-diphenoxyphenyl)pyridine (dPhOppyH$_2$)[10] were prepared according to reported methods. (Piperidinomethyl)polystyrene (~3.5 mmol/g base loading) was purchased from Merck.

The experimental setup for irradiations with green light is similar to previously reported designs.[11] It consisted of a 2 L crystallizing dish with a commercial RGB LED strip working in green color (Inspire LEDFLEXI KIT, model 2128KS-5-RGBW, 2.5 m, 4.8 W/m, $\lambda_{max}$ = 516 nm) fixed to the inner wall, in which a 50 mL Schlenk tube containing the reaction mixture and a magnetic stir bar was introduced. The entire system was covered with aluminium foil and placed on a magnetic stirrer. The radiant flux impacting on the Schlenk tube was *ca.* 800 mW based on an optical power measurement. The emission spectrum of the LEDs is shown in Figure S1.

Irradiations with violet light ($\lambda_{max}$ = 405 nm) were performed in flat-bottom, 20 mL Carius tubes (∅ = 20 mm) made of borosilicate glass and fitted with a PTFE vacuum stopcock, which were placed on top of individual LED emitters (LED Engin LuxiGen™ LZ1-10UB0R-00U8) fixed to an aluminium heat sink and cooled by a fan. The radiant flux at the bottom of the tube was *ca.* 1380 mW based on technical specifications. The emission spectrum of the LEDs is shown in Figure S1.

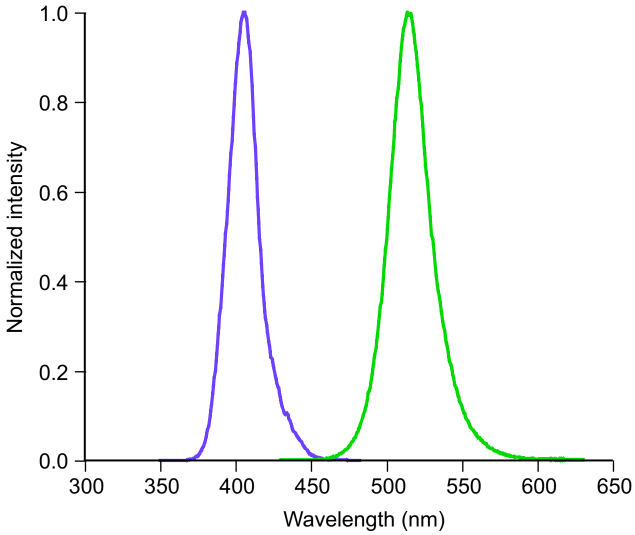


**Figure S1.** Emission spectra of the violet (left) and green (right) LED emitters employed for irradiations.

## 1.2. Spectroscopic and analytical methods

NMR spectra were recorded on Bruker Avance 300, 400, or 600 MHz spectrometers at 298 K. Chemical shifts are referred to residual signals of non-deuterated solvent and are given in ppm downfield from tetramethylsilane. Electronic absorption spectra were recorded on a Perkin-Elmer Lambda 750S spectrophotometer. Elemental analyses were carried out with a LECO CHNS-932 microanalyzer. Infrared spectra were recorded on a Jasco FT/IR-4600 spectrometer equipped with an attenuated total reflectance (ATR) module.

## 1.3. Synthesis of $(Bu_4N)_2[Pt_2Cl_6]$

The published method for the synthesis of this compound involves heating of an aqueous solution of $K_2PtCl_4$ and $Bu_4NCl$ (1:3 molar ratio) at 80 °C.[12] We present an alternative procedure.

To a suspension of $PtCl_2$ (1.00 g, 3.76 mmol) in $CH_2Cl_2$ (100 mL) was added $Bu_4NCl$ (1.31 g, 4.71 mmol) and the mixture was stirred at room temperature in the dark until a clear pink-red solution was obtained (48-72 h), which was filtered through Celite. The solvent was removed under reduced pressure and the resulting oily residue was stirred in EtOH (20 mL) until a pink-red crystalline solid formed. The product was collected by filtration, washed with EtOH (3 × 10 mL) and $Et_2O$ (3 × 5 mL) and vacuum-dried. Yield: 1.52 g, 75%. Elemental analysis calcd for $C_{32}H_{72}Cl_6N_2Pt_2$: C, 35.33; H, 6.67; N, 2.58; found: C, 35.23; H, 6.86; N, 2.41. IR (ATR, $cm^{-1}$): ν(Pt–Cl) = 337, 315 (coincide with the reported frequencies[12]).

## 1.4. Synthesis of 2-(4,4''-dimethyl-[1,1':3',1''-terphenyl]-5'-yl)pyridine ($dmtppyH_2$)

This compound was prepared following the reported procedure for the analogous 2-([1,1':3',1''-terphenyl]-5'-yl)pyridine,[13] from 2-(3,5-dichlorophenyl)pyridine (740 mg, 3.30 mmol), *p*-tolylboronic acid (1.40 g, 10.30 mmol), $K_3PO_4$ (4.2 g, 19.80 mmol), $[Pd(OAc)_2]$ (37 mg, 0.16 mmol) and S-Phos (136 mg, 0.33 mmol) in a mixture of THF/$H_2O$ (3:1 v/v, 20 mL). Purification was achieved by column chromatography on silica gel (*n*-hexane/ethyl acetate, 9:1). White solid. Yield: 804 mg, 73%. $^1H$ NMR (600 MHz, $CD_2Cl_2$): δ 8.72 (ddd, $J_{H-H}$ = 4.8, 1.9, 0.9 Hz, 1H), 8.21 (d, $J_{H-H}$ = 1.7 Hz, 2H), 7.88 (dt, $J_{H-H}$ = 8.0, 1.1 Hz, 1H), 7.86 (t, $J_{H-H}$ = 1.7 Hz, 1H), 7.81 (ddd, $J_{H-H}$ = 8.0, 7.4, 1.8 Hz, 1H), 7.70–7.61 (m, 4H), 7.34–7.30 (m, 4H), 7.29 (ddd, $J_{H-H}$ = 7.4, 4.8, 1.1 Hz, 1H), 2.42 (s, 6H). $^{13}C\{^1H\}$ APT NMR (151 MHz, $CD_2Cl_2$): 157.5 (C), 150.1 (CH), 142.4 (2C), 140.8 (C), 138.5 (2C), 137.9 (2C), 137.2 (CH), 129.9 (4CH), 127.5 (4CH), 126.5 (CH), 124.6 (2CH), 122.8 (CH), 121.0 (CH), 21.3 ($2CH_3$). Elemental analysis calcd for $C_{25}H_{21}N$: C, 89.51; H, 6.31; N, 4.18; found: C, 89.68; H, 6.36; N, 4.10.

## 1.5. Photochemical synthesis of monocyclometalated complexes $Bu_4N[PtCl_2(C^\wedge N)]$ (1) and $[Pt(C^\wedge N)(acac)]$ (2)

**General procedure**. A Schlenk tube was charged with $(Bu_4N)_2[Pt_2Cl_6]$ (50 mg, 0.046 mmol), the appropriate HC^N ligand (0.11 mmol), (piperidinomethyl)polystyrene (53 mg, ~0.186 mmol) and acetone (20 mL). The mixture was irradiated with green light (516 nm) for 48 (ligands **a-f** and **i**) or 72 h (ligands **g** and **h**) under vigorous stirring and filtered through Celite, and the solvent was removed under reduced pressure. In the cases of ligands **a-f**, treatment of the residue with $CH_2Cl_2$ (2 mL) and addition of $Et_2O$ (10 mL) led to the precipitation of a yellow or orange solid. The mother liquor was decanted and the solid was washed with $Et_2O$ (3 × 3 mL) and vacuum-dried to give **1a-f**. In the cases of ligands **g-i**, the corresponding $Bu_4N[PtCl_2(C^\wedge N)]$ salts could not be precipitated as solids. Instead, the residue was suspended in MeOH (2 mL), a solution of Na(acac) (28 mg, 0.23 mmol) in MeOH (3 mL) was added and the mixture was stirred for 30 min. The solvent was removed under reduced pressure, the residue was passed through a short chromatography column using $CH_2Cl_2$ as the eluent and the first orange or yellow band was collected. Evaporation of the solvent afforded **2g-i**.

**$Bu_4N[PtCl_2(ppy)]$ (1a).** Yellow solid. Yield: 40 mg, 66%. The $^1H$ NMR data agree with those reported in the literature.[14]

**$Bu_4N[PtCl_2(tpy)]$ (1b).** Yellow solid. Yield: 43 mg, 69%. This complex has been previously reported, but the characterization data were not given.[15] $^1H$ NMR (600 MHz, $CD_2Cl_2$): δ 9.86 (ddd with satellites, $J_{H-H}$ = 6.0, 1.7, 0.8 Hz, $J_{H-Pt}$ = 40 Hz, 1H), 7.82 (s with satellites, $J_{H-Pt}$ = 40 Hz, 1H), 7.76 (ddd, $J_{H-H}$ = 8.0, 7.4, 1.6 Hz, 1H), 7.52 (br d, $J_{H-H}$ = 7.8 Hz, 1H), 7.26 (d, $J_{H-H}$ = 7.8 Hz, 1H), 7.01 (ddd, $J_{H-H}$ = 7.4, 5.9, 1.5 Hz, 1H),

6.85 (ddd, $J_{H-H}$ = 7.8, 1.9, 0.8 Hz, 1H), 3.29 – 3.20 (m, 8H), 2.33 (s, 3H), 1.60 – 1.52 (m, 8H), 1.32 (h, $J_{H-H}$ = 7.4 Hz, 8H), 0.90 (t, $J_{H-H}$ = 7.4 Hz, 12H). $^{13}C\{^1H\}$ APT NMR (151 MHz, $CD_2Cl_2$): δ 168.0 (C), 150.3 (CH), 142.2 (C), 141.3 (C), 139.7 (C), 137.4 (CH), 134.2 ($J_{C-Pt}$ ~ 51 Hz, CH), 123.5 (CH), 123.1 ($J_{C-Pt}$ ~ 41 Hz, CH), 121.4 ($J_{C-Pt}$ ~ 30 Hz, CH), 117.9 ($J_{C-Pt}$ ~ 44 Hz, CH), 59.3 ($4CH_2$), 24.5 ($4CH_2$), 22.0 ($CH_3$), 20.0 ($4CH_2$), 13.8 ($4CH_3$). Elemental analysis calcd for $C_{28}H_{46}Cl_2N_2Pt$: C, 49.70; H, 6.85; N, 4.14. Found: C, 49.70; H, 6.76; N, 4.18.

**$Bu_4N[PtCl_2(dfppy)]$ (1c).** Yellow solid. Yield: 40 mg, 62%. This complex has been previously mentioned in the literature, but it was not isolated.[16] $^1H$ NMR (600 MHz, $CD_2Cl_2$): δ 9.95 (ddd with satellites, $J_{H-H}$ = 5.9, 1.7, 0.8 Hz, $J_{H-Pt}$ = 40 Hz, 1H), 7.95 (br d, $J_{H-H}$ = 8.2 Hz, 1H), 7.86 – 7.80 (m, 1H), 7.64 (dd with satellites, $J_{H-F}$ = 9.9 Hz, $J_{H-H}$ = 2.5 Hz, $J_{H-Pt}$ = 54 Hz, 1H), 7.10 (ddd, $J_{H-H}$ = 7.5, 5.9, 1.6 Hz, 1H), 6.56 (ddd, $J_{H-F}$ = 12.6, 9.0 Hz, $J_{H-H}$ = 2.5 Hz, 1H), 3.28–3.18 (m, 8H), 1.63–1.53 (m, 8H), 1.34 (h, $J_{H-H}$ = 7.4 Hz, 8H), 0.91 (t, $J_{H-H}$ = 7.4 Hz, 12H). $^{13}C\{^1H\}$ APT NMR (151 MHz, $CD_2Cl_2$): δ 164.6 (d, $J_{C-F}$ ~ 6 Hz, C), 163.1 (dd, $J_{C-F}$ = 254, 13 Hz, C), 160.3 (dd, $J_{C-F}$ = 258, 13 Hz, C), 150.5 (CH), 145.5 (d, $J_{C-F}$ = 7 Hz, C), 138.1 (CH), 128.6 (C), 122.0 (CH), 121.9 (d, $J_{C-F}$ ~ 20 Hz, CH), 115.3 (d, $J_{C-F}$ = 19 Hz, $J_{C-Pt}$ ~ 50 Hz, CH), 98.1 (t, $J_{C-F}$ = 27 Hz, CH), 59.3 ($4CH_2$), 24.5 ($4CH_2$), 20.0 ($4CH_2$), 13.8 ($4CH_3$). $^{19}F$ NMR (282 MHz, $CD_2Cl_2$): –108.67 (br dt with satellites, $J_{F-H}$ ~ 9.4 Hz, $J_{F-F}$ ~ 9.4 Hz, $J_{F-Pt}$ = 60 Hz, 1F), –113.07 (br dd with satellites, $J_{F-H}$ ~ 12.3 Hz, $J_{F-F}$ = 9.2 Hz, $J_{H-Pt}$ ~ 46 Hz, 1F). Elemental analysis calcd for $C_{27}H_{42}Cl_2F_2N_2Pt$: C, 46.42; H, 6.06; N, 4.01. Found: C, 46.46; H, 6.24; N, 4.03.

**$Bu_4N[PtCl_2(thpy)]$ (1d).** Orange solid. Yield: 39 mg, 63%. The $^1H$ NMR data agree with those reported in the literature.[17]

**$Bu_4N[PtCl_2(Bppy)]$ (1e).** Yellowish orange solid. Yield: 49 mg, 59%. $^1H$ NMR (600 MHz, $CD_2Cl_2$): δ 9.94 (d with satellites, $J_{H-H}$ = 1.6 Hz, $J_{H-Pt}$ ~ 38 Hz, 1H), 8.02 (dd with satellites, $J_{H-H}$ = 7.8, 1.3 Hz, $J_{H-Pt}$ ~ 34 Hz, 1H), 7.78 (dd, $J_{H-H}$ = 8.0, 1.6 Hz, 1H), 7.55 (d, $J_{H-H}$ = 8.0 Hz, 1H), 7.43 (dd, $J_{H-H}$ = 7.8, 1.5 Hz, 1H), 7.08 (td, $J_{H-H}$ = 7.4, 1.5 Hz, 1H), 7.03 (td, $J_{H-H}$ = 7.4, 1.4 Hz, 1H), 6.87 (s, 4H), 3.29–3.15 (m, 8H), 2.32 (s, 6H), 2.04 (s, 12H), 1.59–1.52 (m, 8H), 1.33 (h, $J_{H-H}$ = 7.4 Hz, 8H), 0.90 (t, $J_{H-H}$ = 7.3 Hz, 12H). $^{13}C$ APT NMR (151 MHz, $CD_2Cl_2$): δ 170.2 (C), 158.4 (CH), 145.5 (CH), 144.6 (C), 143.9 (C), 141.3 (4C), 141.0 (C), 139.7 (2C), 137.7 (2C), 133.7 (CH), 130.4 (CH), 128.8 (4CH), 124.1 (CH), 122.6 (CH), 117.6 (CH), 59.3 ($4CH_2$), 24.5 ($4CH_2$), 23.8 ($4CH_3$), 21.4 ($2CH_3$), 20.1 ($4CH_2$), 13.8 ($4CH_3$). Elemental analysis calcd for $C_{45}H_{65}BCl_2N_2Pt$: C, 59.34; H, 7.19; N, 3.08. Found: C, 59.30; H, 7.25; N, 3.02.

**$Bu_4N[PtCl_2(flpy)]$ (1f).** Yellow solid. Yield: 47 mg, 65%. $^1H$ NMR (600 MHz, $CD_2Cl_2$): δ 9.90 (ddd with satellites, $J_{H-H}$ = 5.9, 1.6, 0.7 Hz, $J_{H-Pt}$ ~ 35 Hz, 1H), 8.43 (s with satellites, $J_{H-Pt}$ ~ 37 Hz, 1H), 7.80 (ddd, $J_{H-H}$ = 8.1, 7.3, 1.6 Hz, 1H), 7.77 (ddd, $J_{H-H}$ = 7.2, 1.5, 0.8 Hz, 1H), 7.66 (br d, $J_{H-H}$ = 7.9 Hz, 1H), 7.46 (s, 1H), 7.43 (ddd, $J_{H-H}$ = 7.2, 1.5, 0.8 Hz, 1H), 7.31 (td, $J_{H-H}$ = 7.3, 1.5 Hz, 1H), 7.28 (td, $J_{H-H}$ = 7.3, 1.5 Hz, 1H), 7.04 (ddd, $J_{H-H}$ = 7.4, 5.9, 1.5 Hz, 1H), 3.31–3.19 (m, 8H), 1.58–1.50 (m, 8H), 1.49 (s, 6H), 1.29 (h, $J_{H-H}$ = 7.4 Hz, 8H), 0.86 (t, $J_{H-H}$ = 7.4 Hz, 12H). $^{13}C$ APT NMR (151 MHz, $CD_2Cl_2$): δ 167.9 (C), 155.0 (C), 150.5 (CH), 148.4 (C), 143.8 (C), 140.7 (C), 140.4 (C), 140.2 (C), 137.4 (CH), 127.5 (CH), 127.2 (CH), 124.8 (CH), 122.8 (CH), 121.6 (CH), 121.0 (CH), 118.2 (CH), 117.4 (CH), 59.4 ($4CH_2$), 46.7 (C), 27.6 ($2CH_3$), 24.6 ($4CH_2$), 20.1 ($4CH_2$), 13.8 ($4CH_3$). Elemental analysis calcd for $C_{36}H_{52}Cl_2N_2Pt$: C, 55.52; H, 6.73; N, 3.60. Found: C, 55.53; H, 6.70; N, 3.45.

**[Pt(pq)(acac)] (2g).** Orange solid. Yield: 14 mg, 31%. The $^1H$ NMR data agree with those reported in the literature.[18]

**[Pt(bzq)(acac] (2h).** Yellow solid. Yield: 15 mg, 35%. The $^1H$ NMR data agree with those reported in the literature.[19]

**[Pt(bzopy)(acac)] (2i).** Yellow solid. Yield: 22 mg, 50%. $^1$H NMR (600 MHz, $CD_2Cl_2$): δ 9.12 (ddt with satellites, $J_{H-H}$ = 5.9, 1.5, 0.5 Hz, $J_{H-Pt}$ = 44 Hz, 1H), 8.18 (ddt, $J_{H-H}$ = 7.9, 1.7, 0.6 Hz, 1H), 8.06 (dddd, $J_{H-H}$ = 7.9, 7.6, 1.5, 0.5 Hz, 1H), 7.78 (ddd, $J_{H-H}$ = 7.8, 1.6, 0.5 Hz, 1H), 7.63 (ddd with satellites, $J_{H-H}$ = 7.8, 1.2, 0.5 Hz, $J_{H-Pt}$ = 40 Hz, 1H), 7.49 (dddd, $J_{H-H}$ = 7.6, 5.9, 1.7, 0.5 Hz, 1H), 7.27 (ddd, $J_{H-H}$ = 7.8, 7.1, 1.6 Hz, 1H), 7.12 (ddd, $J_{H-H}$ = 7.7, 7.1, 1.2 Hz, 1H), 5.56 (s, 1H), 2.00 (s, 3H), 1.99 (s, 3H). $^{13}$C{$^1$H} APT NMR (151 MHz, $CD_2Cl_2$): δ 192.1 (CO), 187.1 (CO), 185.2 (CO), 153.6 (C), 152.1 (CH), 138.6 (CH), 135.7 (CH), 133.3 (2C), 130.9 ($J_{C-Pt}$ ~ 48 Hz, CH), 127.7 ($J_{C-Pt}$ ~ 27 Hz, CH), 127.6 (CH), 126.5 (CH), 124.5 (CH), 102.4 (CH), 28.2 ($CH_3$), 27.1 ($CH_3$). Elemental analysis calcd for $C_{17}H_{15}NO_3Pt$: C, 42.86; H, 3.17; N, 2.94. Found: C, 42.82; H, 3.45; N, 3.10.

## 1.6. Photochemical synthesis of bis-cyclometalated complexes *cis*-[Pt(C^N)₂] (3)

**General procedure.** A Carius tube was charged with [$PtCl_2(NCPh)_2$] (75 mg, 0.16 mmol), the HC^N ligand (0.44 mmol), $Na_2CO_3$ (47 mg, 0.44 mmol) and acetone (10 mL). The mixture was irradiated with violet light for 24 (ligands **a-c**, **f**) or 48 h (ligands **d**, **g**) under vigorous stirring. The solvent was removed under reduced pressure and the residue was passed through a short silica gel chromatography column using $CH_2Cl_2$ as the eluent. The yellow or orange band was collected. Evaporation of the solvent gave complexes **3a**, **3b**, **3d**, **3f** and **3g**. Complex **3c** precipitated from the reaction mixture after irradiation and was collected by filtration, washed with $H_2O$ (3 × 3 mL), MeOH (3 × 3 mL) and $Et_2O$ (3 × 3 mL), and vacuum-dried.

***cis*-[Pt(ppy)$_2$] (3a).** Red-orange solid. Yield: 43 mg, 54%. The $^1$H NMR data agree with those reported in the literature.[20]

***cis*-[Pt(tpy)$_2$] (3b).** Yellow solid. Yield: 48 mg, 57%. The $^1$H NMR data agree with those reported in the literature.[11]

***cis*-[Pt(dfppy)$_2$] (3c).** Dark red solid. Yield: 48 mg, 52%. The $^1$H NMR data agree with those reported in the literature.[11]

***cis*-[Pt(thpy)$_2$] (3d).** Red solid. Yield: 40 mg, 49%. The $^1$H NMR data agree with those reported in the literature.[20]

***cis*-[Pt(flpy)$_2$] (3f).** Yellowish orange solid. Yield: 50 mg, 43%. $^1$H NMR (600 MHz, $CD_2Cl_2$): δ 8.84 (br d with satellites, $J_{H-H}$ ~ 5.1 Hz, $J_{H-Pt}$ ~ 11 Hz, 2H), 8.62 (s with satellites, $J_{H-Pt}$ = 52 Hz, 2H), 8.02 (br d, $J_{H-H}$ ~ 8.2 Hz, 2H), 7.96 (ddd, $J_{H-H}$ = 8.1, 7.2, 1.5 Hz, 2H), 7.84 – 7.79 (m, 2H), 7.78 (s, 2H), 7.52 – 7.46 (m, 2H), 7.37 – 7.29 (m, 6H), 1.59 (s, 12H). $^{13}$C{$^1$H} APT NMR (151 MHz, $CD_2Cl_2$): δ 166.7 (2C), 155.0 (2C), 149.3 (2C), 148.4 (2C), 148.2 (2CH), 146.0 (2C), 141.3 (2C), 140.2 (2C), 138.3 (2CH), 129.0 ($J_{C-Pt}$~ 99 Hz, 2CH), 127.6 (2CH), 127.5 (2CH), 123.0 (2CH), 122.3 (2CH), 120.6 (2CH), 119.7 (2CH), 118.2 ($J_{C-Pt}$ ~ 34 Hz, 2CH), 46.9 (2C), 27.6 (4$CH_3$). Elemental analysis calcd for $C_{40}H_{32}N_2Pt$: C, 65.30; H, 4.38; N, 3.81. Found: C, 65.26; H, 4.40; N, 4.02.

***cis*-[Pt(pq)$_2$] (3g).** Red solid. Yield: 23 mg, 24%. The $^1$H NMR data agree with those reported in the literature.[21]

## 1.7. Photochemical synthesis of Pt(II) complexes with terdentade ligands

**[PtCl(pbpy)] (4). Method A.** A Carius tube was charged with $(Bu_4N)_2[Pt_2Cl_6]$ (100 mg, 0.09 mmol), pbpyH (47 mg, 0.20 mmol), $Na_2CO_3$ (24 mg, 0.22 mmol) and acetone (10 mL). The mixture was irradiated with

violet light for 16 h under vigorous stirring, resulting in an orange suspension. The solvent was removed under reduced pressure, the residue was treated with $CH_2Cl_2$ (20 mL) and the suspension was filtered through Celite. The filtrate was concentrated (3 mL) and $Et_2O$ (15 mL) was added, whereupon an orange solid precipitated. The mother liquor was decanted and the solid was washed with $Et_2O$ (3 × 4 mL) and vacuum-dried to give **4**. Yield: 77 mg, 91%. **Method B.** A Carius tube was charged with [$PtCl_2(NCPh)_2$] (75 mg, 0.16 mmol), pbpyH (40 mg, 0.17 mmol), $Na_2CO_3$ (24 mg, 0.22 mmol) and acetone (10 mL). The mixture was irradiated with violet light for 16 h under vigorous stirring, resulting in a brownish orange suspension. The solvent was removed under reduced pressure and the residue was chromatographed on silica gel using a $CH_2Cl_2$/MeOH mixture as the eluent (gradient of methanol was increased from 0 to 10%). The orange band was collected and the solvent was removed under reduced pressure to give **4**. Yield: 34 mg, 46%. The $^1$H NMR data agree with those reported in the literature.[22]

**[PtCl(dfdpyb)] (5). Method A.** A Carius tube was charged with $(Bu_4N)_2[Pt_2Cl_6]$ (100 mg, 0.09 mmol), dfdpybH (54 mg, 0.20 mmol), $Na_2CO_3$ (24 mg, 0.22 mmol) and acetone (10 mL). The mixture was irradiated with violet light for 16 h under vigorous stirring, resulting in a yellow suspension. The solvent was removed under reduced pressure, the residue was treated with $CH_2Cl_2$ (30 mL) and the suspension was filtered through Celite. Partial evaporation of the filtrate (4 mL) and addition of $Et_2O$ (15 mL) led to the precipitation of a yellow solid. The mother liquor was decanted and the solid was washed with $Et_2O$ (3 × 5 mL) and MeOH (2 × 3 mL) and vacuum-dried to give **5** as an orange solid. Yield: 84 mg, 92%. **Method B.** The same procedure was followed using [$PtCl_2(NCPh)_2$] (75 mg, 0.16 mmol), dfdpybH (46 mg, 0.17 mmol), $Na_2CO_3$ (24 mg, 0.22 mmol) and acetone (10 mL). Yield: 56 mg, 71%. The $^1$H NMR data agree with those reported in the literature.[9]

**$Bu_4N$[PtCl(dmtppy)] (6).** A Carius tube was charged with $(Bu_4N)_2[Pt_2Cl_6]$ (100 mg, 0.09 mmol), dmtppy$H_2$ (68 mg, 0.20 mmol), $Na_2CO_3$ (39 mg, 0.37 mmol) and acetone (10 mL). The mixture was irradiated with violet light for 18 h under vigorous stirring, resulting in a yellow suspension. The solvent was removed under reduced pressure, the residue was treated with $CH_2Cl_2$ (15 mL) and the suspension was filtered through Celite. Partial evaporation of the filtrate (1 mL) and addition of $Et_2O$ (5 mL) led to the precipitation of a yellow solid, which was collected by filtration, washed with $Et_2O$ (4 × 5 mL) and vacuum-dried to give **6**. Yield: 130 mg, 88%. $^1$H NMR (600 MHz, $CD_2Cl_2$): δ 9.27 (ddd with satellites, $J_{H\text{-}H}$ = 5.4, 1.7, 0.8 Hz, $J_{H\text{-}Pt}$ = 18 Hz, 1H), 7.89 (ddd, $J_{H\text{-}H}$ = 7.9, 7.3, 1.7 Hz, 1H), 7.77 (d, $J_{H\text{-}H}$ = 7.8 Hz, 1H), 7.63 (dd with satellites, $J_{H\text{-}H}$ = 1.4, 0.6 Hz, $J_{H\text{-}Pt}$ = 62 Hz, 1H), 7.59-7.55 (m, 2H), 7.43 (d with satellites, $J_{H\text{-}H}$ = 1.5 Hz, $J_{H\text{-}Pt}$ = 10 Hz, 1H), 7.37 (d with satellites, $J_{H\text{-}H}$ = 1.5 Hz, $J_{H\text{-}Pt}$ = 14 Hz, 1H), 7.29 (ddd, $J_{H\text{-}H}$ = 7.4, 5.4, 1.3 Hz, 1H), 7.26–7.23 (m, 2H), 7.21 (d, $J_{H\text{-}H}$ = 7.5 Hz, 1H), 6.76 (ddd, $J_{H\text{-}H}$ = 7.5, 1.9, 0.8 Hz, 1H), 2.94–2.89 (m, 8H), 2.37 (s, 3H), 2.28 (s, 3H), 1.30–1.23 (m, 8H), 1.11 (h, $J_{H\text{-}H}$ = 7.3 Hz, 8H), 0.78 (t, $J_{H\text{-}H}$ = 7.3 Hz, 12H). $^{13}C\{^1H\}$ NMR data could not be recorded because the complex decomposes in $CH_2Cl_2$ within a few hours. Elemental analysis calcd for $C_{41}H_{55}ClN_2Pt$: C, 61.06; H, 6.87; N, 3.47; found: C, 61.13; H, 6.86; N, 3.53.

**[Pt(dPhOppy)(γ-pic)] (8).** A Carius tube was charged with $(Bu_4N)_2[Pt_2Cl_6]$ (100 mg, 0.09 mmol), dPhOppy$H_2$ (69 mg, 0.20 mmol), $Na_2CO_3$ (47 mg, 0.44 mmol) and acetone (10 mL). The mixture was irradiated with violet light under vigorous stirring for 18 h and the resulting suspension was concentrated (5 mL). γ-Picoline (72 µL, 0.74 mmol) was then added and the suspension was stirred for 30 min. The solvent was removed under reduced pressure, the residue was treated with $CH_2Cl_2$ (10 mL) and the suspension was filtered through a silica pad. The yellow filtrate was evaporated to dryness and the residue was treated with MeOH (5 mL) to afford a yellow precipitate, which was collected by filtration, washed with MeOH (4 × 2 mL) and vacuum-dried to give **8**. Yield: 70 mg, 61%. $^1$H NMR (600 MHz, $CD_2Cl_2$): δ 8.91 (dd with satellites, $J_{H\text{-}H}$ = 6.4, 1.6 Hz, $J_{H\text{-}Pt}$ ~ 21 Hz, 2H), 7.81 (ddd, $J_{H\text{-}H}$ = 8.6, 7.1, 1.6 Hz, 1H), 7.77 (br d, $J_{H\text{-}H}$ ~ 7.8 Hz, 1H),

7.48 (ddd with satellites, $J_{H-H}$ = 5.5, 1.6, 0.8 Hz, $J_{H-Pt}$ ~ 16 Hz, 1H), 7.42 (ddd, $J_{H-H}$ = 5.5, 1.6, 0.8 Hz, 2H), 7.38 – 7.30 (m, 2H), 7.20 (d, $J_{H-H}$ = 2.2 Hz, 1H), 7.16 – 7.05 (m, 4H), 7.04 (ddd, $J_{H-H}$ = 7.1, 5.6, 1.5 Hz, 1H), 7.01 (ddd, $J_{H-H}$ = 8.2, 6.8, 1.7 Hz, 1H), 6.81 (d with satellites, $J_{H-H}$ = 2.2 Hz, $J_{H-Pt}$ = 27 Hz, 1H), 6.68 (dd with satellites, $J_{H-H}$ = 7.7, 1.7 Hz, $J_{H-Pt}$ ~ 72 Hz, 1H), 6.59 (ddd, $J_{H-H}$ = 7.9, 6.9, 1.5 Hz, 1H), 2.53 (s, 3H). $^{13}C\{^1H\}$ APT NMR (151 MHz, $CD_2Cl_2$): δ 164.4 (C), 158.8 (C), 154.3 (C), 154.1 (C), 153.2 (C), 152.0 (2CH), 150.6 (C), 148.0 (C), 147.5 (CH), 138.3 (2CH), 130.0 (2CH), 128.0 (2CH), 124.4 (CH), 123.5 (CH), 122.9 (CH), 120.6 ($J_{C-Pt}$ ~ 50 Hz, CH), 119.8 ($J_{C-Pt}$ ~ 21 Hz, CH), 118.3 (2CH), 117.1 (C), 116.7 (C), 116.1 ($J_{C-Pt}$ ~ 28 Hz, CH), 110.8 ($J_{C-Pt}$ ~ 38 Hz, CH), 109.6 ($J_{C-Pt}$ ~ 21 Hz, CH), 21.6 ($CH_3$). Elemental analysis calcd for $C_{29}H_{22}N_2O_2Pt$: C, 55.68; H, 3.54; N, 4.48; found: C, 55.68; H, 3.51; N, 4.32.

**[Pt(dPhOppy)(NCPh)] (10).** A Carius tube was charged with $[PtCl_2(NCPh)_2]$ (100 mg, 0.21 mmol), dPhOppy$H_2$ (79 mg, 0.23 mmol), $Na_2CO_3$ (45 mg, 0.42 mmol) and acetone (10 mL). The mixture was irradiated with violet light for 16 h under vigorous stirring, resulting in a yellow suspension. The solvent was removed under reduced pressure and the residue was suspended in benzonitrile (1 mL). $CH_2Cl_2$ (10 mL) was then added and the suspension was filtered through a silica pad. The yellow filtrate was concentrated (2 mL) and MeOH (20 mL) was added, whereupon a yellow solid precipitated, which was collected by filtration, washed with MeOH (4 × 5 mL) and vacuum-dried to give **10**. Yield: 82 mg, 61%. $^1H$ NMR (600 MHz, $CD_2Cl_2$): δ 8.86 (ddd, $J_{H-H}$ = 5.5, 1.7, 0.8 Hz, 1H), 8.09 (dd with satellites, $J_{H-H}$ = 7.7, 1.8 Hz, $J_{H-Pt}$ ~ 72 Hz, 1H), 8.01 (dd, $J_{H-H}$ = 8.4, 1.2 Hz, 2H), 7.90 (ddd, $J_{H-H}$ = 8.1, 7.4, 1.7 Hz, 1H), 7.85 (tt, $J_{H-H}$ = 7.7, 1.3 Hz, 1H), 7.81 (br d, $J_{H-H}$ ~ 8.2 Hz, 1H), 7.67 (ddt, $J_{H-H}$ = 8.9, 7.6, 1.3 Hz, 2H), 7.39 – 7.33 (m, 2H), 7.32 (ddd, $J_{H-H}$ = 7.1, 5.5, 1.3 Hz, 1H), 7.22 (d, $J_{H-H}$ = 2.3 Hz, 1H), 7.18 – 7.04 (m, 5H), 6.88 (ddd, $J_{H-H}$ = 7.7, 6.0, 2.4 Hz, 1H), 6.83 (d with satellites, $J_{H-H}$ = 2.2 Hz, $J_{H-Pt}$ = 27 Hz, 1H). $^{13}C\{^1H\}$ APT NMR (151 MHz, $CD_2Cl_2$): δ 163.6 (C), 158.4 (C), 155.0 (C), 153.2 (C), 152.7 (C), 148.4 (C), 148.3 (CH), 139.9 (CH), 138.8 (CH), 135.0 (CH), 133.2 (2CH), 130.2 (2CH), 130.1 (2CH), 125.0 (CH), 123.9 (CH), 123.2 (CH), 121.0 ($J_{C-Pt}$ ~ 48 Hz, CH), 119.9 ($J_{C-Pt}$ ~ 16 Hz, CH), 119.3 (C), 118.6 (2CH), 116.8 (C), 116.1 (CH), 114.9 (C), 111.3 (C), 110.5 ($J_{C-Pt}$ ~ 38 Hz, CH), 109.6 ($J_{C-Pt}$ ~ 21 Hz, CH). Elemental analysis calcd for $C_{30}H_{20}N_2O_2Pt$: C, 56.69; H, 3.17; N, 4.41; found: C, 56.68; H, 3.12; N, 4.36.

**[Pt(dmtppy)(γ-pic)] (11).** A Carius tube was charged with $[PtCl_2(NCPh)_2]$ (50 mg, 0.11 mmol), dmtppy$H_2$ (38 mg, 0.11 mmol), $Na_2CO_3$ (23 mg, 0.22 mmol) and acetone (10 mL). The mixture was irradiated with violet light under vigorous stirring for 18 h and the resulting suspension was concentrated (5 mL). γ-Picoline (43 μL, 0.44 mmol) was then added and the mixture was stirred for 30 min. The solvent was removed under reduced pressure, the residue was treated with $CH_2Cl_2$ (10 mL) and the suspension was filtered through a silica pad. The yellow filtrate was concentrated (1 mL) and $Et_2O$ (10 mL) was added, whereupon a yellow solid precipitated, which was collected by filtration, washed with $Et_2O$ (3 × 2 mL) and vacuum-dried to give **11**. Yield: 44 mg, 67%. $^1H$ NMR (600 MHz, $CD_2Cl_2$): δ 8.84 (d with satellites, $J_{H-H}$ = 6.3 Hz, $J_{H-Pt}$ = 22 Hz 2H), 7.97 (ddd with satellites, $J_{H-H}$ = 5.4, 1.7, 0.9 Hz, $J_{H-Pt}$ = 18 Hz, 1H), 7.91 (td, $J_{H-H}$ = 7.6, 1.7 Hz, 1H), 7.85 (d, $J_{H-H}$ = 7.8 Hz, 1H), 7.60 (d, $J_{H-H}$ = 8.1 Hz, 2H), 7.47 (d, $J_{H-H}$ = 1.5 Hz, $J_{H-Pt}$ ~ 7 Hz, 1H), 7.44 (d, $J_{H-H}$ = 1.5 Hz, $J_{H-Pt}$ ~ 11 Hz, 1H), 7.41 (dd, $J_{H-H}$ = 6.3, 0.8 Hz, 2H), 7.29 (d, $J_{H-H}$ = 7.5 Hz, 1H), 7.27 (d, $J_{H-H}$ = 8.0 Hz, 2H), 7.16 (ddd, $J_{H-H}$ = 6.9, 5.4, 1.5 Hz, 1H), 6.79 (ddd, $J_{H-H}$ = 7.6, 1.9, 0.8 Hz, 1H), 6.48 (s with satellites, $J_{H-Pt}$ = 64 Hz, 1H), 2.50 (s, 3H), 2.40 (s, 3H), 2.16 (s, 3H). $^{13}C\{^1H\}$ APT NMR (151 MHz, $CD_2Cl_2$): δ 166.9 (C), 164.1 (C), 154.7 (C), 154.3 (C) 151.6 (2CH), 149.8 (C), 148.4 (CH), 147.9 ($J_{C-Pt}$ = 1243 Hz, C), 141.9 ($J_{C-Pt}$ = 50 Hz, C), 140.6 (C), 138.5 (CH), 136.6 (C), 136.5 (C), 135.7 ($J_{C-Pt}$ = 65 Hz, C), 135.2 ($J_{C-Pt}$ = 36 Hz, CH), 129.8 (2CH), 127.4 (2CH), 127.0 (2CH), 124.6 (CH), 123.0 (CH), 120.3 ($J_{C-Pt}$ = 74 Hz, CH), 119.5 (CH), 118.9 ($J_{C-Pt}$ = 58 Hz, CH), 118.5 ($J_{C-Pt}$ = 41 Hz, CH), 21.7 ($CH_3$), 21.5 ($CH_3$), 21.2 ($CH_3$). Elemental analysis calcd for $C_{31}H_{26}N_2Pt$: C, 59.90; H, 4.22; N, 4.51; found: C, 59.80; H, 4.38; N, 4.48.

## 1.8. Reaction between $[PtCl_2(NCPh)_2]$ and 1,3-di(2-pyridyl)benzene (dpybH) under photochemical conditions

A Carius tube was charged with $[PtCl_2(NCPh)_2]$ (75 mg, 0.16 mmol), dpybH (36 µL, 0.17 mmol), $Na_2CO_3$ (24 mg, 0.22 mmol) and acetone (10 mL). The mixture was irradiated with violet light under vigorous stirring for 16 h, resulting in a yellow suspension. The solvent was evaporated under reduced pressure and the yellow solid was washed with water (3 × 5 mL), MeOH (2 × 5 mL), acetone (3 × 5 mL) and $Et_2O$ (4 × 5 mL) by means of succesive centrifugation and decantation steps, and then vacuum-dried. Yield: 66 mg, 90%. Elemental analysis calcd for $C_{16}H_{11}ClN_2Pt$: C, 41.61; H, 2.40; N, 6.07; found: C, 41.69; H, 2.38; N, 5.86.

**Additional discussion.** The elemental analysis of the material obtained from the photochemical reaction between $[PtCl_2(NCPh)_2]$ and dpybH was consistent with the desired product, [PtCl(dpyb)]. The solid was almost completely insoluble in most organic solvents and the $^1$H NMR spectrum of the very small fraction that dissolved in $CDCl_3$ showed the presence of free dpybH and a very small relative proportion of [PtCl(dpyb)]. The material was soluble in DMSO-$d_6$ but the $^1$H NMR spectrum in this solvent (Figure S2) showed resonances of several unidentified species, some of them broad, along with free dpybH and trace amounts of [PtCl(dpyb)].[8] The most likely explanation of this result is that metalation at the C4/C6 positions of the benzene ring occurs easily under photochemical conditions, leading to an array of different species, possibly including dimers and oligomers with dimetalated dpyb and N-coordinated dpybH at some of the metal centers. We note that a complicated mixture was also reported by Williams *et al.* to result from the reaction of $K_2[PtCl_4]$ with dpybH in MeCN/$H_2O$ at reflux temperature for 3 days.[23]

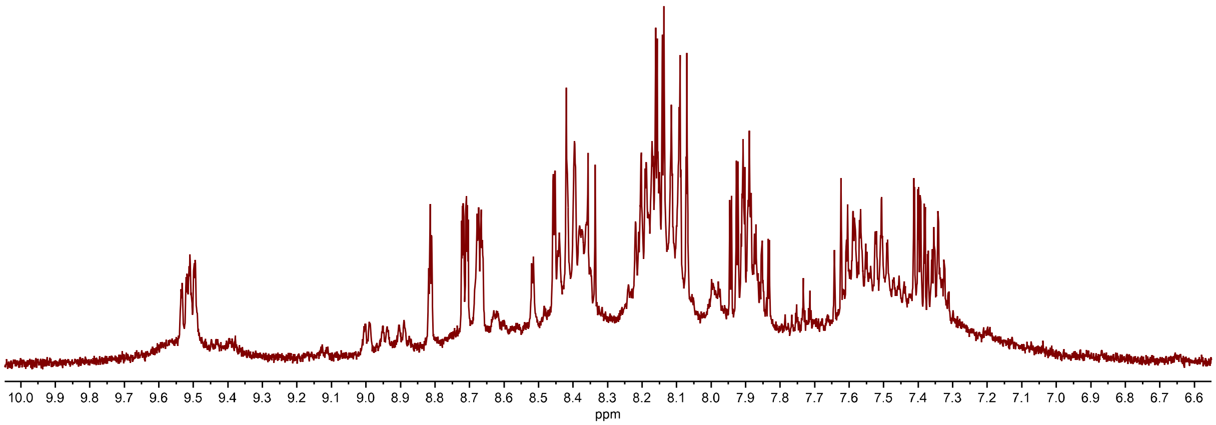


**Figure S2.** $^1$H NMR spectrum (aromatic region) of the product mixture resulting from the reaction between $[PtCl_2(NCPh)_2]$ and dpybH under photochemical conditions (DMSO-$d_6$, 400 MHz).

## 1.9. Thermal cycloplatinations of dmtppy$H_2$ and dPhOppy$H_2$

The precursors $(Bu_4N)_2[Pt_2Cl_6]$ and $[PtCl_2(NCPh)_2]$ did not react with dmtppy$H_2$ or dPhOppy$H_2$ in acetone at room temperature in the dark. When a EtOH/$CH_2Cl_2$ mixture was employed as solvent, metalation of dPhOppy$H_2$ was observed at room temperature in the dark using $(Bu_4N)_2[Pt_2Cl_6]$ as precursor, but the yield was 24% after 3 days. By refluxing a mixture of $(Bu_4N)_2[Pt_2Cl_6]$ and dPhOppy$H_2$ in MeCN in the presence of $Na_2CO_3$, a mixture was obtained, which contained only a small proportion of complex **7**. The reaction of $[PtCl_2(NCPh)_2]$ with dPhOppy$H_2$ in toluene at reflux temperature in the presence of $Na_2CO_3$ gave a mixture in which complex **10** was a minor product.

The reactions between $K_2[PtCl_4]$ and dmtppy$H_2$ or dPhOppy$H_2$ in AcOH at reflux temperature led to complex mixtures under an inert atmosphere. In the case of the dmtppy$H_2$, refluxing in AcOH under aerobic conditions produced pure material of a very low solubility, which we postulate as the dimeric Pt(IV) complex $[Pt_2Cl_2(\mu\text{-}Cl)_2(\text{dmtppy})_2]$ (**12**; 51% yield) on the basis of its elemental analysis and its reaction with γ-picoline to give the mononuclear complex $[PtCl_2(\text{dmtppy})(\gamma\text{-picoline})]$ (**13**) (Scheme S1), whose identity was confirmed by an X-ray diffraction analysis (Figure S5).

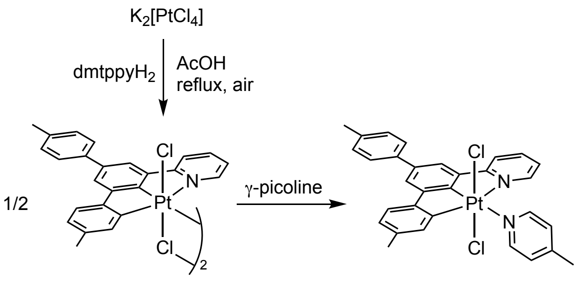


**12** **13**

**Scheme S1.** Thermal synthesis of Pt(IV) complexes with dmtppy.

**$[Pt_2Cl_2(\mu\text{-}Cl)_2(\text{dmtppy})_2]$ (12).** A mixture of $K_2PtCl_4$ (124 mg, 0.30 mmol) and dmtppy$H_2$ (100 mg, 0.30 mmol) in acetic acid (15 mL) was refluxed for 24 h under atmospheric conditions, whereupon an orange suspension was obtained. After cooling to room temperature, the precipitate was collected by filtration, washed with $H_2O$ (2 × 2 mL) and $Et_2O$ (2 × 3 mL) and vacuum-dried to give **12** as a light-orange solid. Yield: 92 mg, 51%. Elemental analysis calcd for $C_{50}H_{38}Cl_4N_2Pt_2$: C, 50.09; H, 3.19; N, 2.34; found: C, 50.22; H, 3.36; N, 2.22. NMR spectra could not be registered for this compound because of its very low solubility in common organic solvents.

**$[PtCl_2(\text{dmtppy})(\gamma\text{-pic})]$ (13).** To a suspension of **12** (50 mg, 0.04 mmol) in $CH_2Cl_2$ (10 mL) was added γ-picoline (19 μL, 0.20 mmol) and the mixture was stirred for 10 min. The resulting clear solution was filtered through Celite and the filtrate was concentrated (1 mL). $Et_2O$ (10 mL) was then added, whereupon a yellow solid precipitated, which was collected by filtration, washed with $Et_2O$ (2 × 3 mL) and vacuum-dried to give **13**. Yield: 49 mg, 85%. $^1$H NMR (600 MHz, $CD_2Cl_2$): δ 9.45-9.41 (m, 2H), 8.19-8.12 (m, 2H), 7.98 (td, $J_{H\text{-}H}$ = 8.0, 7.8, 1.6 Hz, 1H), 7.73 (d, $J_{H\text{-}H}$ = 1.6 Hz, 1H), 7.70 (d, $J_{H\text{-}H}$ = 1.6 Hz, 1H), 7.64 (d, $J_{H\text{-}H}$ = 8.1 Hz, 2H), 7.58 (d, $J_{H\text{-}H}$ = 5.8 Hz, 2H), 7.47 (d, $J_{H\text{-}H}$ = 7.7 Hz, 1H), 7.36-7.31 (m, 3H), 6.94 (dd, $J_{H\text{-}H}$ = 7.6, 0.8 Hz, 1H), 6.53 (s with satellites, $J_{H\text{-}Pt}$ = 24 Hz, 1H), 2.60 (s, 3H), 2.45 (s, 3H), 2.27 (s, 3H). $^{13}$C{$^1$H} APT NMR (151 MHz, $CD_2Cl_2$): δ 162.7 (C), 152.3 (2C), 150.1 (2CH), 148.2 (C), 147.9 (CH), 144.8 (C), 140.3 (C), 139.9 (CH), 139.0 (C), 138.4 (2C), 137.6 (C), 132.2 (C), 131.5 (CH), 129.9 (2CH), 127.7 (2CH), 127.5 (2CH), 126.8 (CH), 124.2 (CH), 122.7 ($J_{C\text{-}Pt}$ ~ 38 Hz, CH), 122.2 ($J_{C\text{-}Pt}$ ~ 32 Hz, CH) 121.7 (CH), 121.2 (CH), 21.8 ($CH_3$), 21.6 ($CH_3$), 21.2 ($CH_3$). Elemental analysis calcd for $C_{31}H_{26}Cl_2N_2Pt$: C, 53.76; H, 3.78; N, 4.05; found: C, 53.71; H, 3.92; N, 3.92.

## 1.10. X-Ray structure determinations

Single crystals suitable for X-ray diffraction were grown by slow liquid-liquid diffusion from $CH_2Cl_2$/*n*-pentane (**8**) or $CH_2Cl_2/Et_2O$ (**11** and **13**). Diffraction data were collected on a Bruker D8 QUEST diffractometer with monochromated Mo-*K*α radiation performing $\varphi$ and $\omega$ scans. The structures were solved by direct methods and refined anisotropically on $F^2$ using the program SHELXL-2018 (G. M. Sheldrick, University of Göttingen).[24,25] Numerical details are presented in Table S1. Methyl hydrogens were included as part of rigid idealized methyl groups allowed to rotate but not tip; other hydrogens were included using a riding model.

**Table S1.** Crystallographic data for **8**, **11** and **13**.

| | **8** | **11** | **13** |
|---|---|---|---|
| formula | $C_{29}H_{22}N_2O_2Pt$ | $C_{31}H_{26}N_2Pt$ | $C_{31}H_{26}Cl_2N_2Pt$ |
| fw | 625.57 | 621.63 | 692.53 |
| $T$ (K) | 100(2) | 100(2) | 100(2) |
| λ | 0.71073 | 0.71073 | 0.71073 |
| cryst syst | orthorhombic | monoclinic | triclinic |
| space group | $P2_12_12_1$ | $P2_1/c$ | P-1 |
| $a$ (Å) | 10.5062(9) | 9.9581(9) | 9.40000(10) |
| $b$ (Å) | 10.9667(9) | 11.8225(9) | 11.4102(2) |
| $c$ (Å) | 19.5268(16) | 20.2486(16) | 13.1956(2) |
| $\alpha$ (°) | 90 | 90 | 74.013(4) |
| $\beta$ (°) | 90 | 99.891(2) | 76.084(5) |
| $\gamma$ (°) | 90 | 90 | 87.710(5) |
| $V$ (Å$^3$) | 2249.8(3) | 2348.4(3) | 1320.11(5) |
| $Z$ | 4 | 4 | 2 |
| $\rho_{calcd}$ (Mg m$^{-3}$) | 1.847 | 1.758 | 1.742 |
| $\mu$ (mm$^{-1}$) | 6.267 | 5.998 | 5.540 |
| R1$^a$ | 0.0190 | 0.0216 | 0.0225 |
| wR2$^b$ | 0.0419 | 0.0502 | 0.0518 |

$^a$R1 = $\Sigma||F_o| - |F_c||/\Sigma|F_o|$ for reflections with $I > 2\sigma(I)$. $^b$wR2 = $[\Sigma[w(F_o^2 - F_c^2)^2/\Sigma[w(F_o^2)^2]]^{0.5}$ for all reflections; $w^{-1} = \sigma^2(F^2) + (aP)^2 + bP$, where $P = (2F_c^2 + F_o^2)/3$ and $a$ and $b$ are constants set by the program.

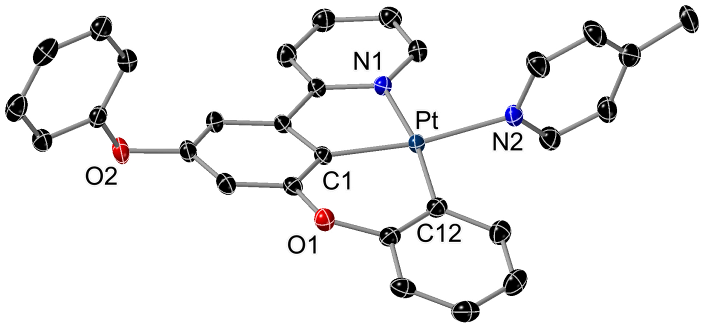


**Figure S3.** Crystal structure of **8** (thermal ellipsoids at 50% probability). Hydrogen atoms are omitted.

**Table S2.** Selected bond distances (Å) and angles (°) for **8**.

| | | | |
|---|---|---|---|
| Pt–C1 | 1.964(4) | Pt–N1 | 2.102(3) |
| Pt–C12 | 1.994(4) | Pt–N2 | 2.114(3) |
| | | | |
| C1–Pt–N1 | 80.77(14) | C12–Pt–N2 | 95.39(16) |
| C1–Pt–C12 | 91.65(17) | C1–Pt–N2 | 171.41(15) |
| Nl–Pt–N2 | 92.65(13) | C12–Pt–N1 | 170.68(15) |

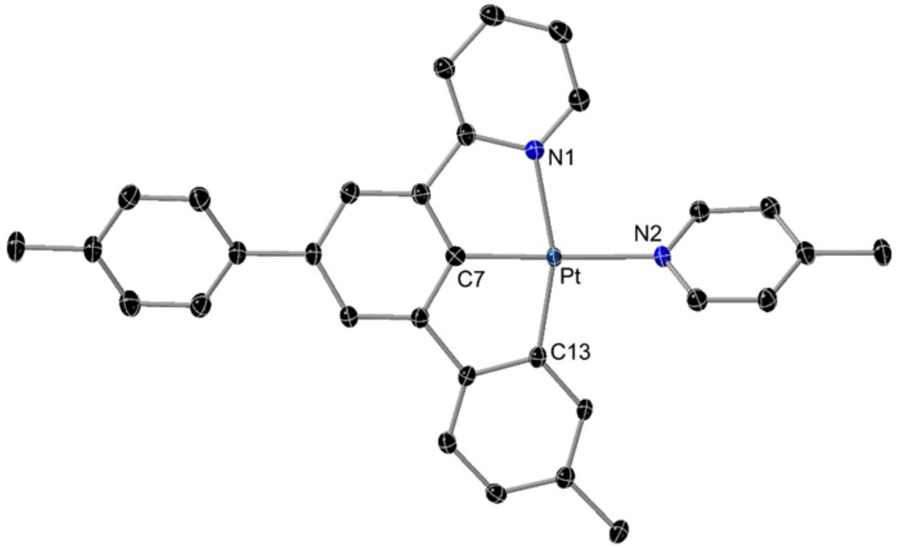


**Figure S4.** Crystal structure of **11** (thermal ellipsoids at 50% probability). Hydrogen atoms are omitted.

**Table S3.** Selected bond distances (Å) and angles (°) for **11**.

| | | | |
|---|---|---|---|
| Pt–C7 | 1.934(3) | Pt–N2 | 2.131(2) |
| Pt–C13 | 2.011(3) | Pt–N1 | 2.140(3) |
| | | | |
| C7–Pt–C13 | 81.23(11) | N1–Pt–C7 | 79.19(11) |
| C13–Pt–N2 | 99.70(10) | C7–Pt–N2 | 178.88(10) |
| N2–Pt–N1 | 99.90(9) | C13–Pt–N1 | 160.26(10) |

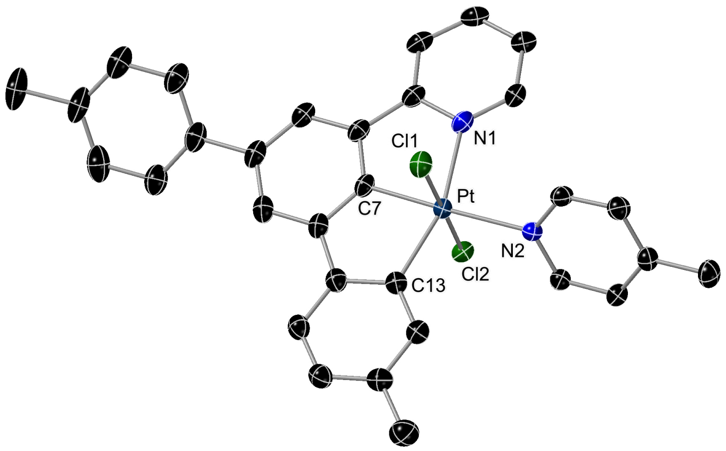


**Figure S5.** Crystal structure of **13** (thermal ellipsoids at 50% probability). Hydrogen atoms are omitted.

**Table S4.** Selected bond distances (Å) and angles (°) for **13**.

| | | | |
|---|---|---|---|
| Pt–C7 | 1.961(3) | Pt–N1 | 2.175(2) |
| Pt–C13 | 2.061(3) | Pt–Cl1 | 2.3095(7) |
| Pt–N2 | 2.208(2) | Pt–Cl2 | 2.3167(6) |
| | | | |
| C7–Pt–N2 | 177.67(10) | N2-Pt-N1 | 98.54(8) |
| C13–Pt–N1 | 160.63(10) | N1-Pt-C7 | 79.24(10) |
| Cl1–Pt–Cl2 | 178.94(2) | C7–Pt–Cl1 | 90.21(8) |
| C7–Pt–C13 | 81.40(11) | N2-Pt-Cl1 | 90.51(6) |
| C13–Pt–N2 | 100.81(10) | | |

## 2. NMR spectra of new compounds

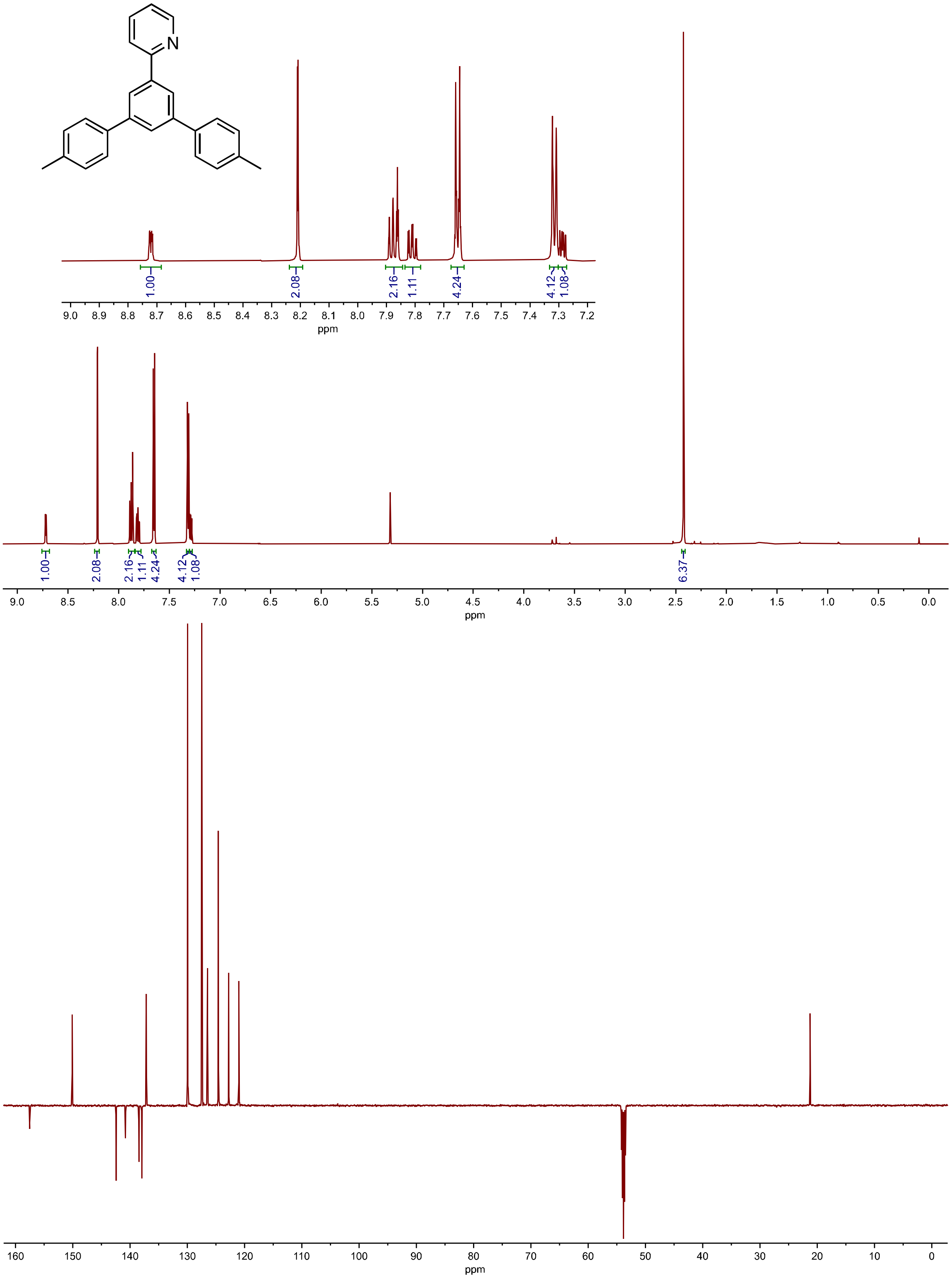


**Figure S6.** $^{1}H$ (top) and $^{13}C\{^{1}H\}$ APT (bottom) NMR spectra of compound dmtppy$H_2$ ($CD_2Cl_2$, 600 and 151 MHz, respectively).

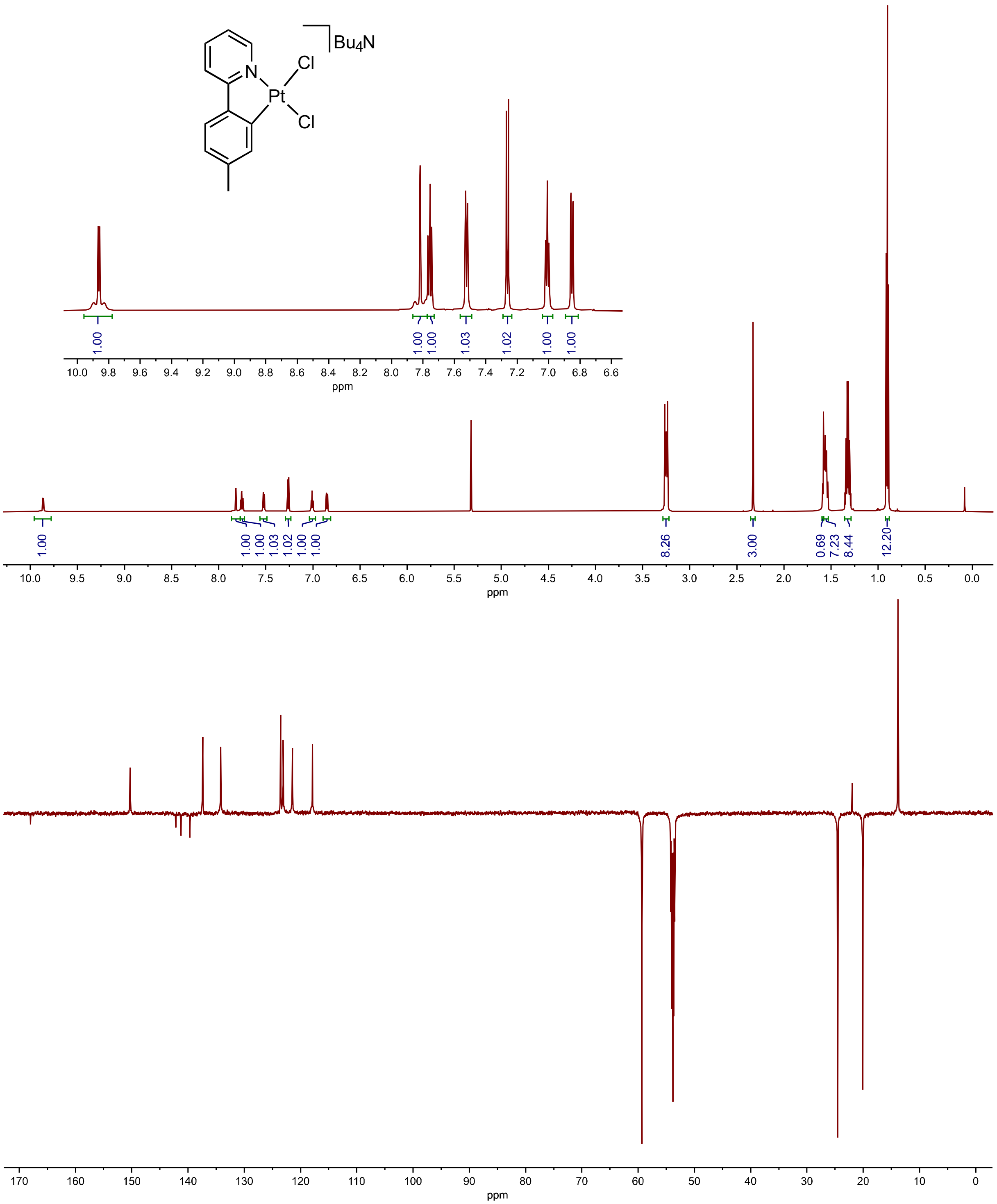


**Figure S7.** $^{1}$H (top) and $^{13}$C{$^{1}$H} APT (bottom) NMR spectra of complex **1b** ($CD_2Cl_2$, 600 and 151 MHz, respectively).

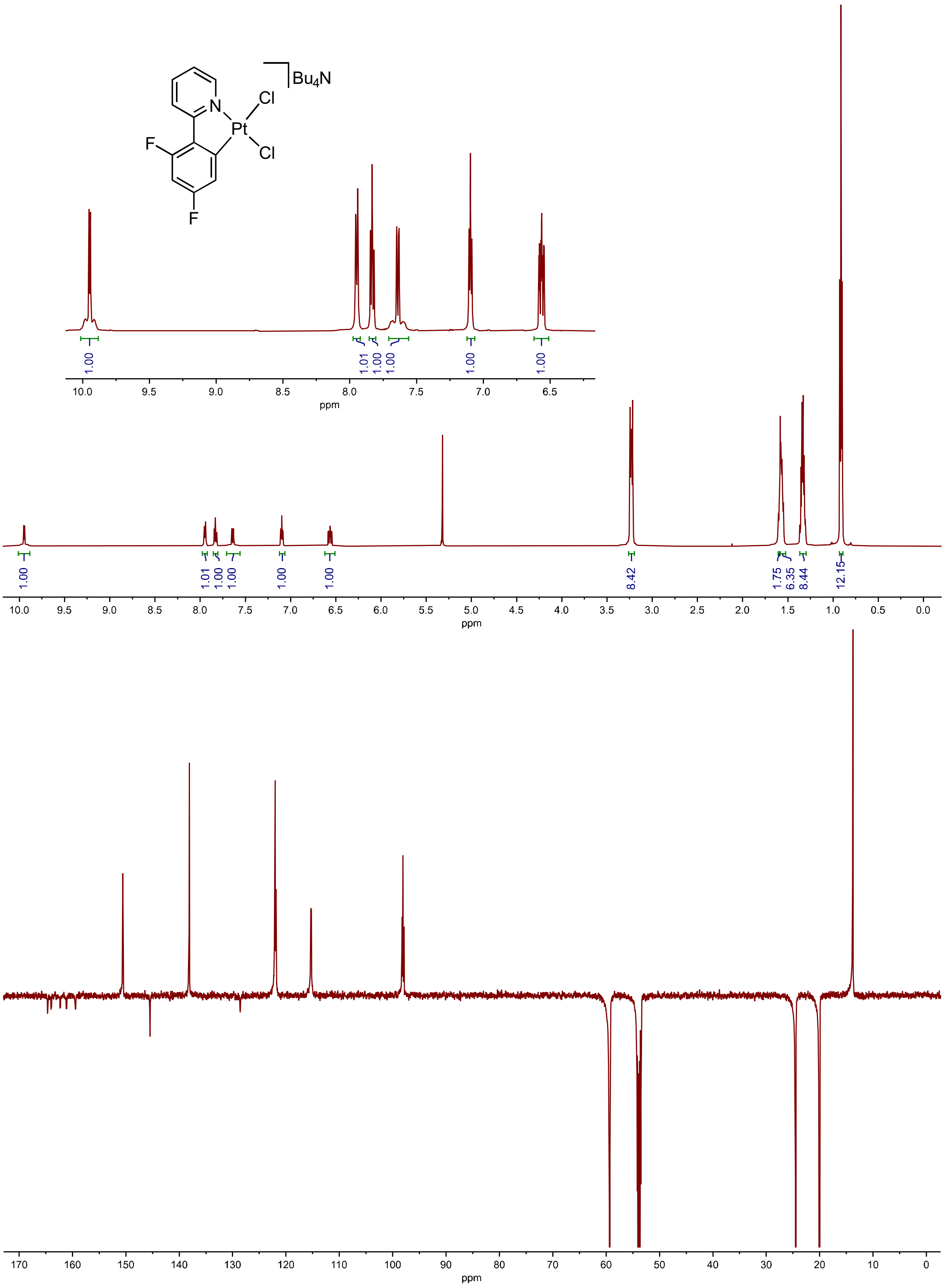


**Figure S8.** $^{1}H$ (top) and $^{13}C\{^{1}H\}$ APT (bottom) NMR spectra of complex **1c** ($CD_2Cl_2$, 600 and 151 MHz, respectively).

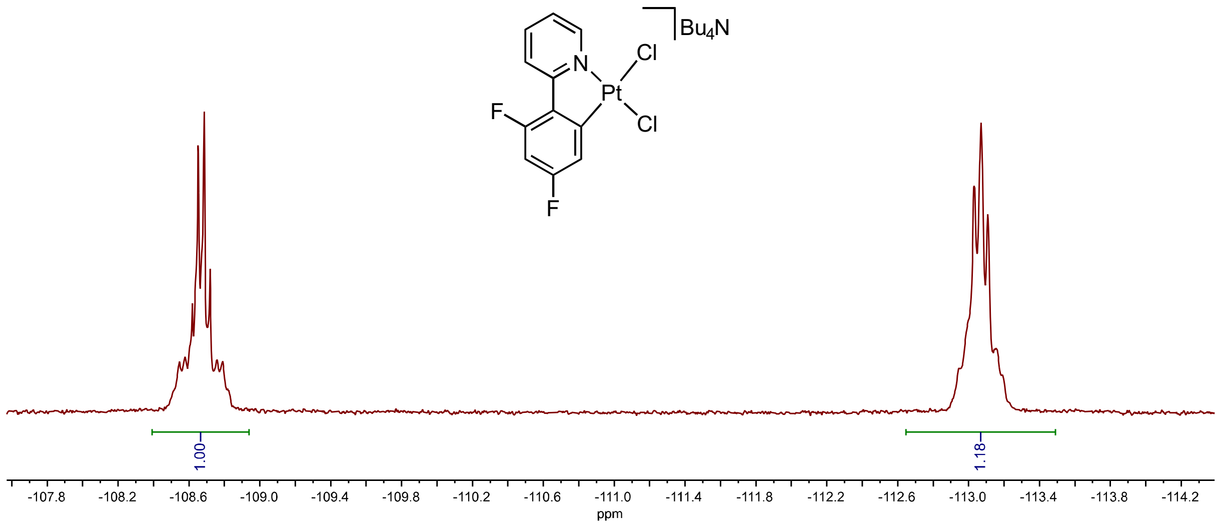


**Figure S9.** $^{19}F$ NMR spectrum of complex **1c** ($CD_2Cl_2$, 282 MHz).

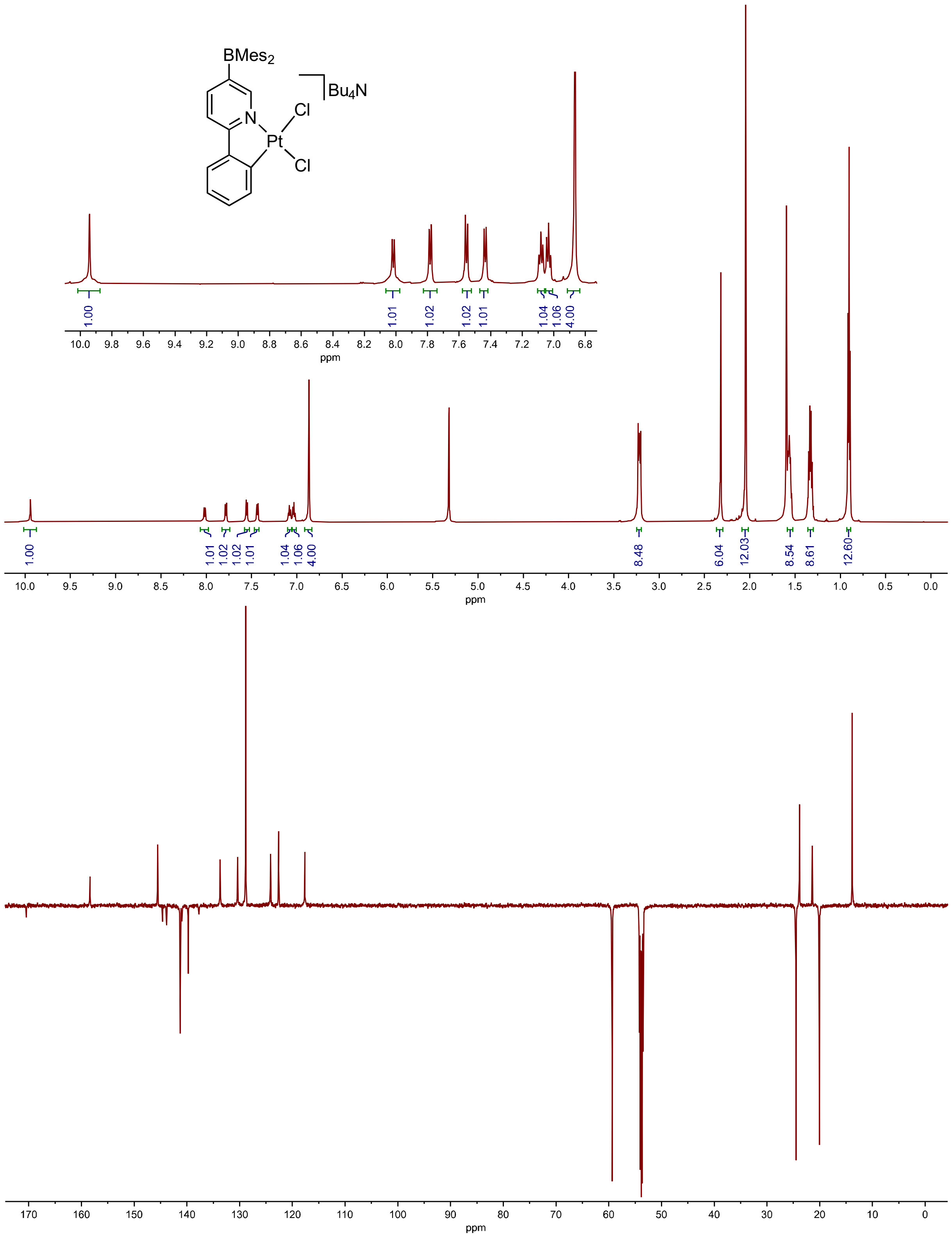


$^1H$ (top) and $^{13}C\{^1H\}$ APT (bottom) NMR spectra of complex **1e** ($CD_2Cl_2$, 600 and 151 MHz, respectively).

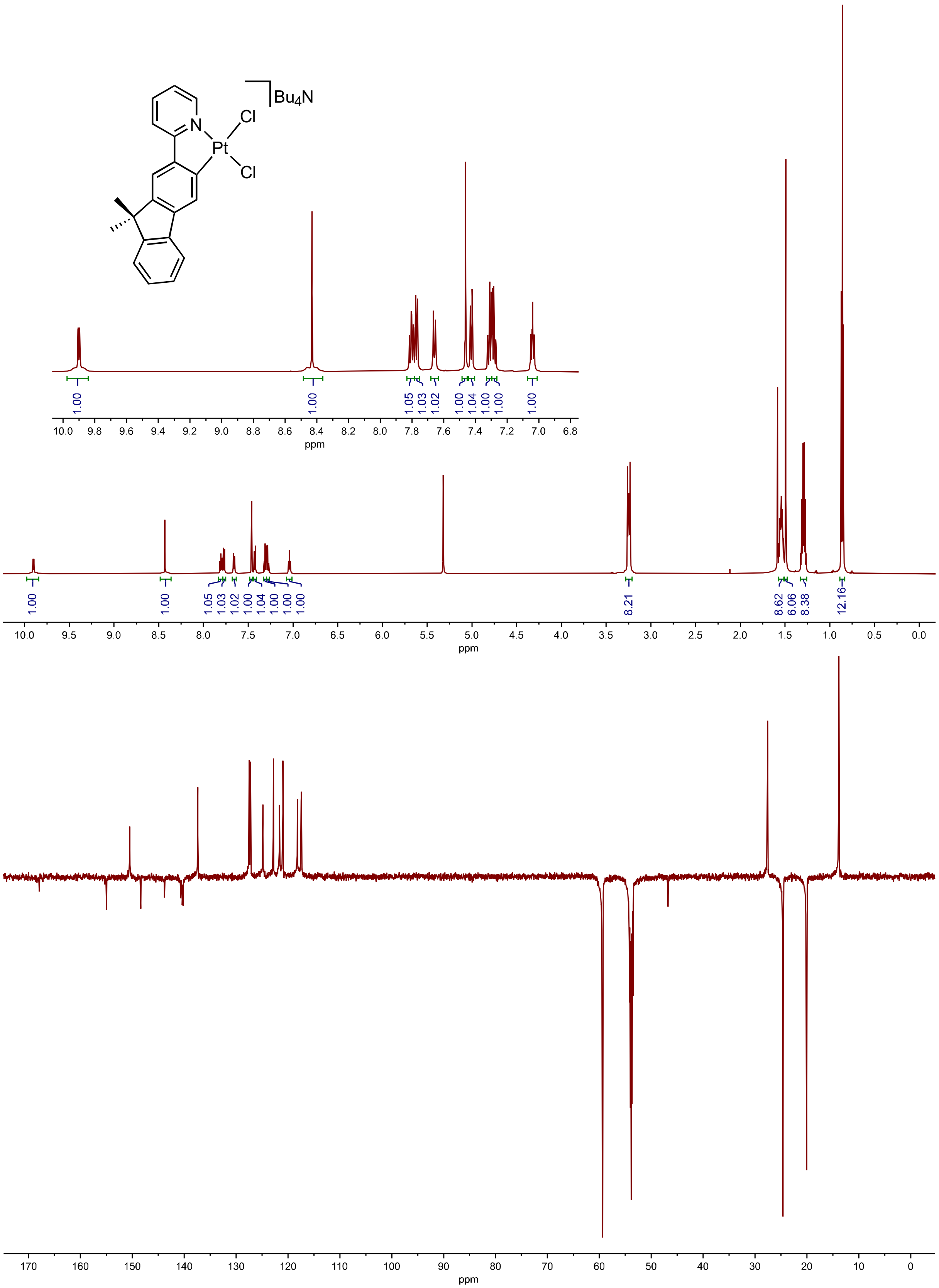


**Figure S10.** $^1H$ (top) and $^{13}C\{^1H\}$ APT (bottom) NMR spectra of complex **1f** ($CD_2Cl_2$, 600 and 151 MHz, respectively).

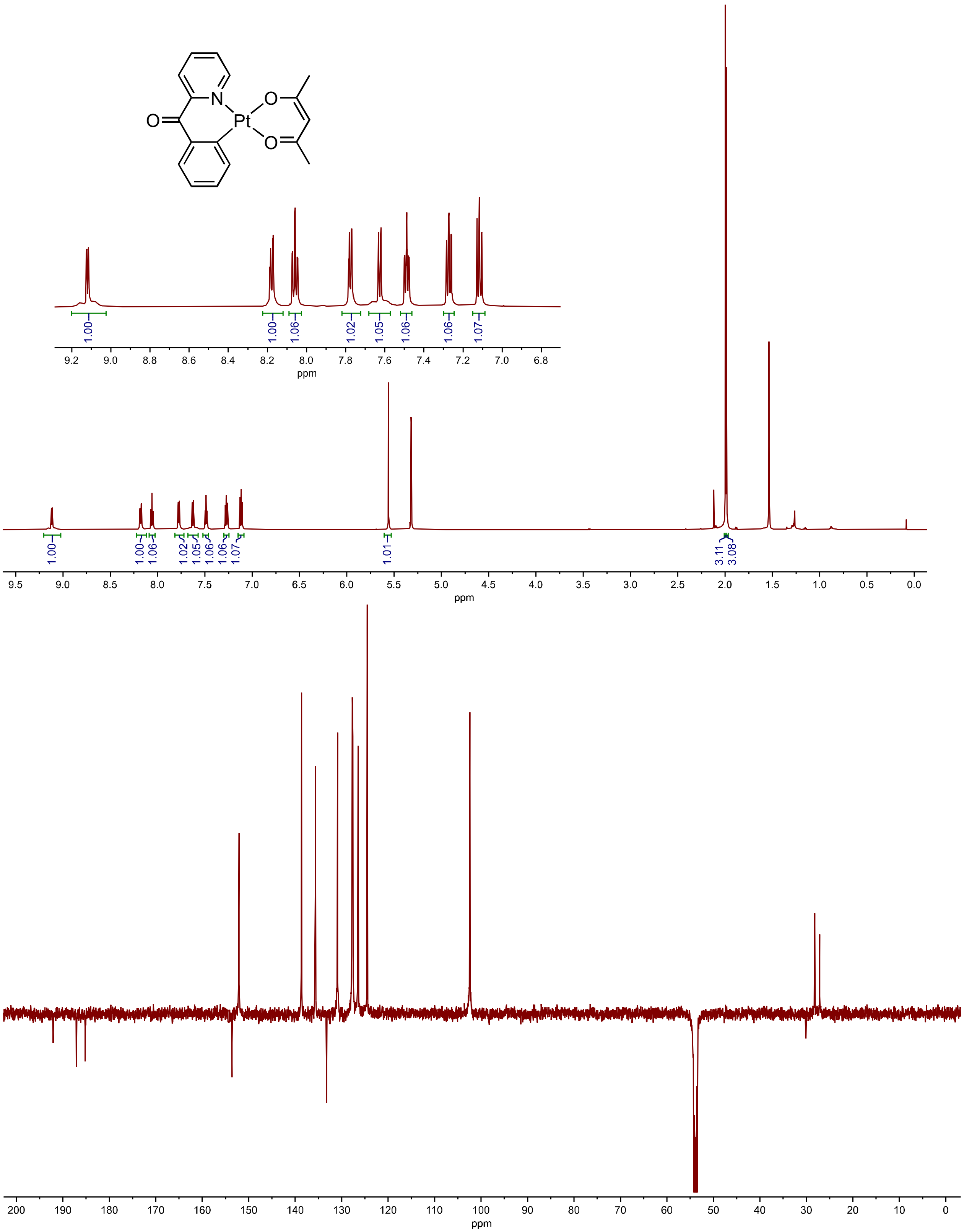


**Figure S11.** $^{1}H$ (top) and $^{13}C\{^{1}H\}$ APT (bottom) NMR spectra of complex **2i** ($CD_2Cl_2$, 600 and 151 MHz, respectively).

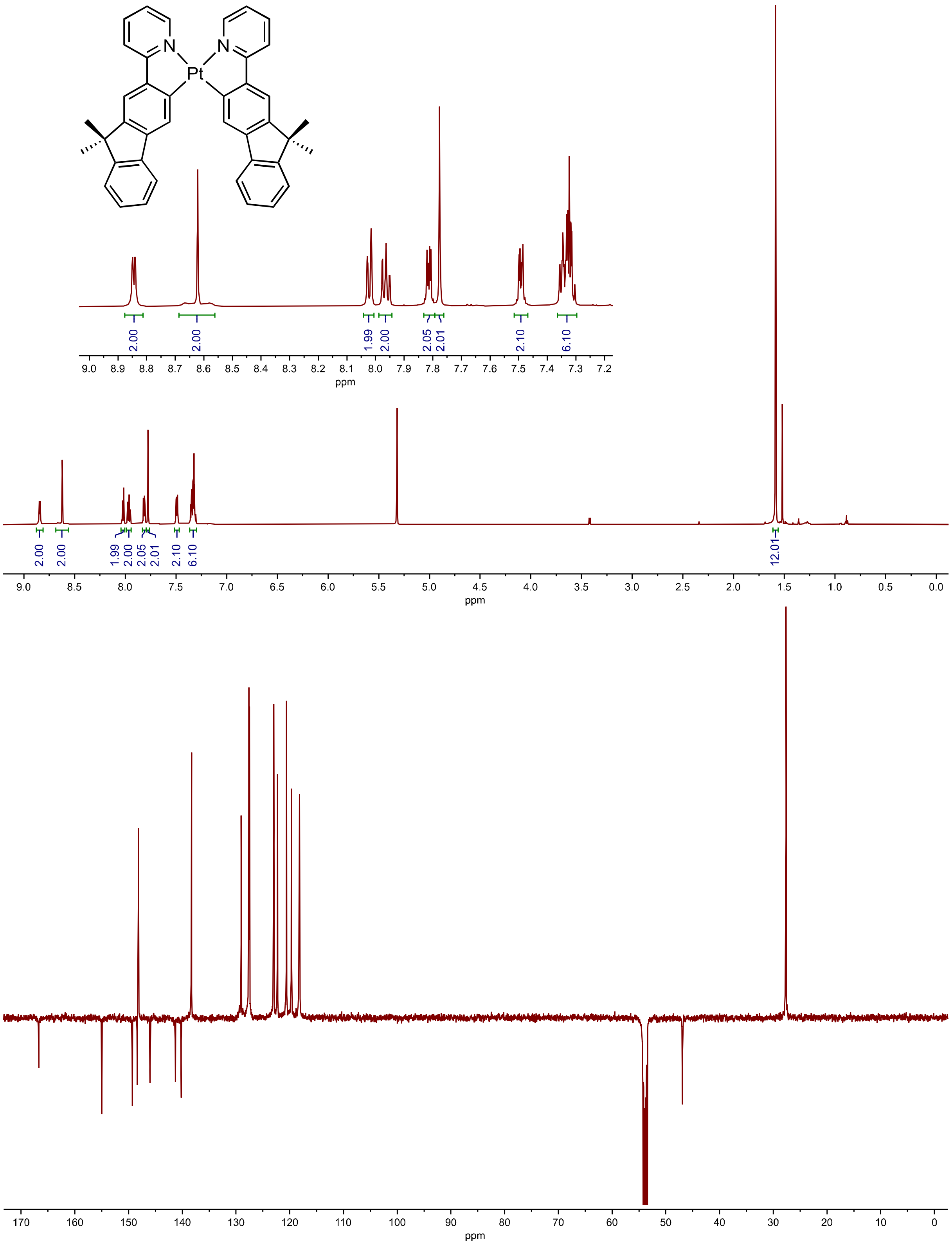


**Figure S12.** $^{1}H$ (top) and $^{13}C\{^{1}H\}$ APT (bottom) NMR spectra of complex **3f** ($CD_2Cl_2$, 600 and 151 MHz, respectively).

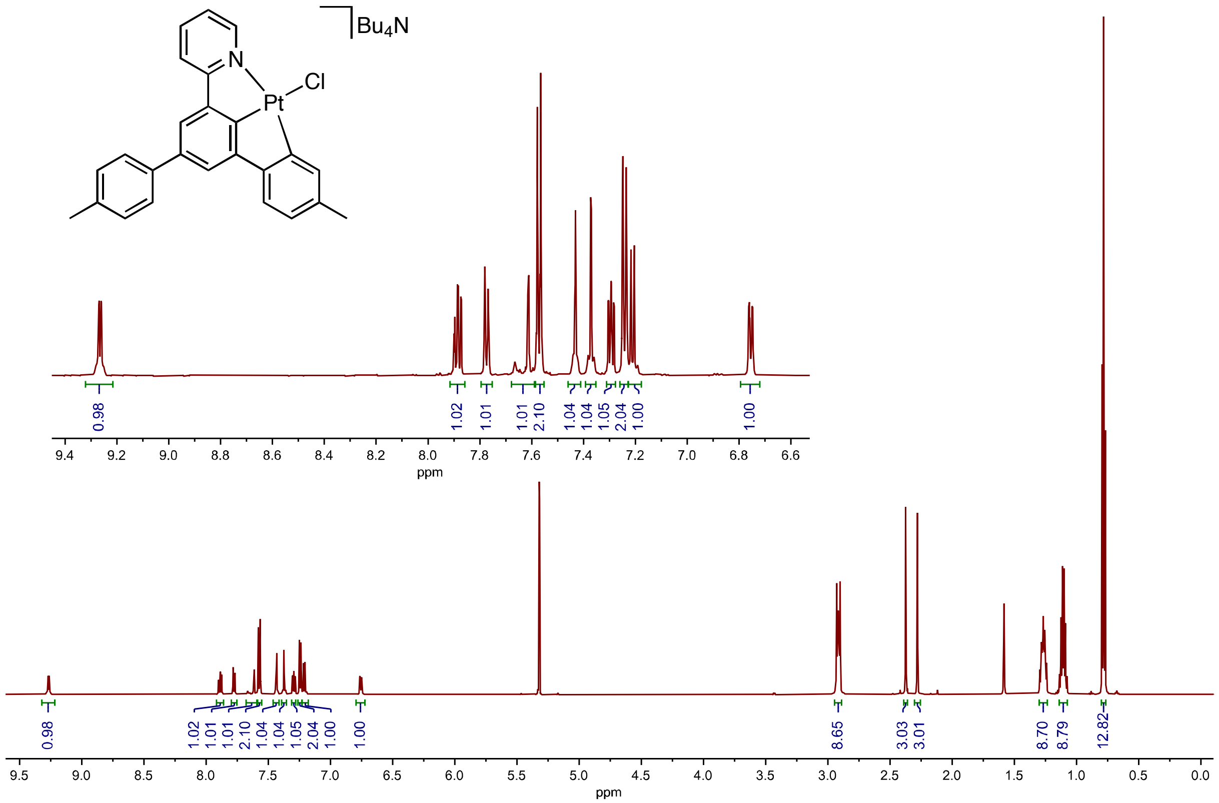

**Figure S13.** $^{1}$H NMR spectrum of complex **6** ($CD_2Cl_2$, 600 MHz).

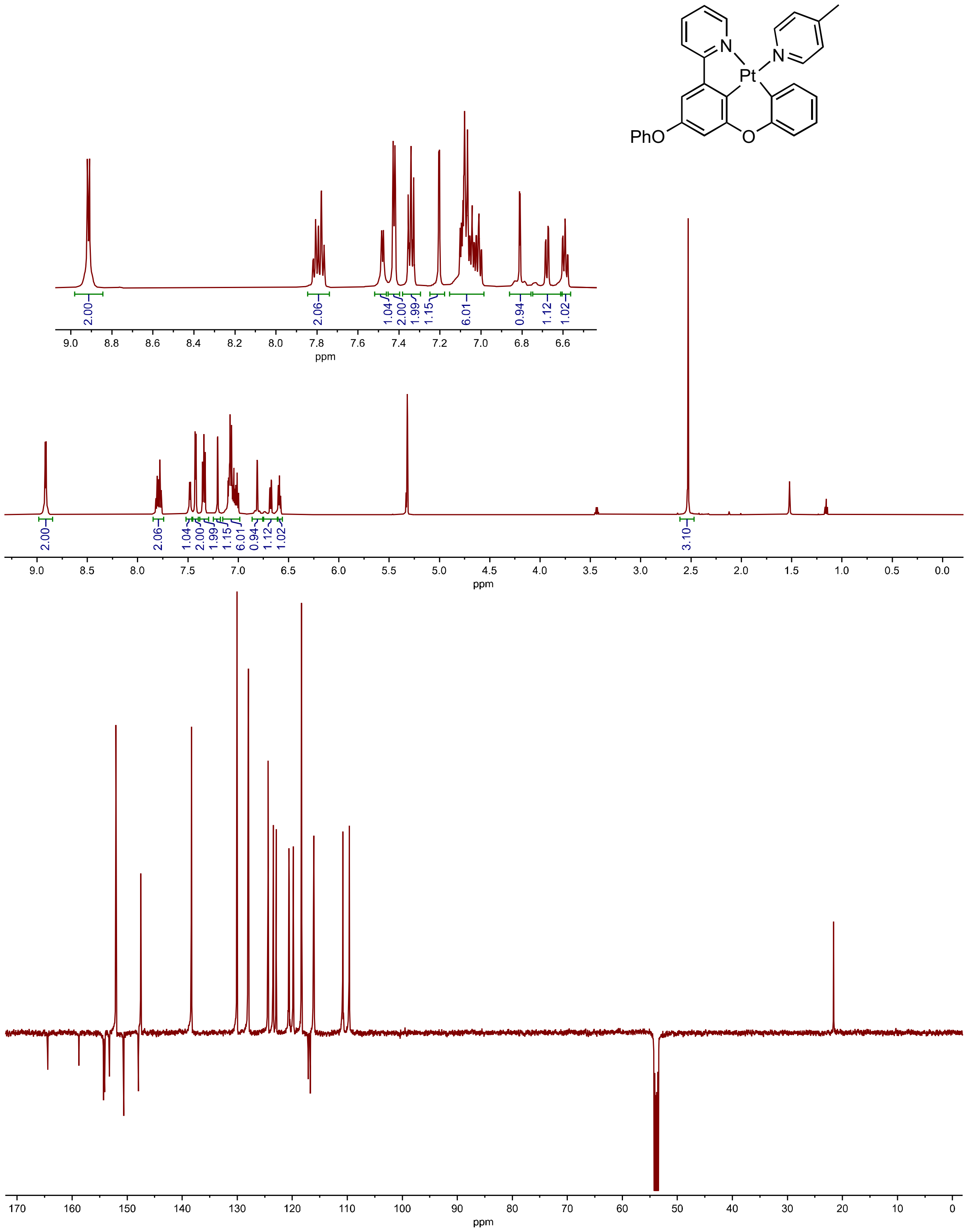


**Figure S14.** $^{1}H$ (top) and $^{13}C\{^{1}H\}$ APT (bottom) NMR spectra of complex **8** ($CD_2Cl_2$, 600 and 151 MHz, respectively).

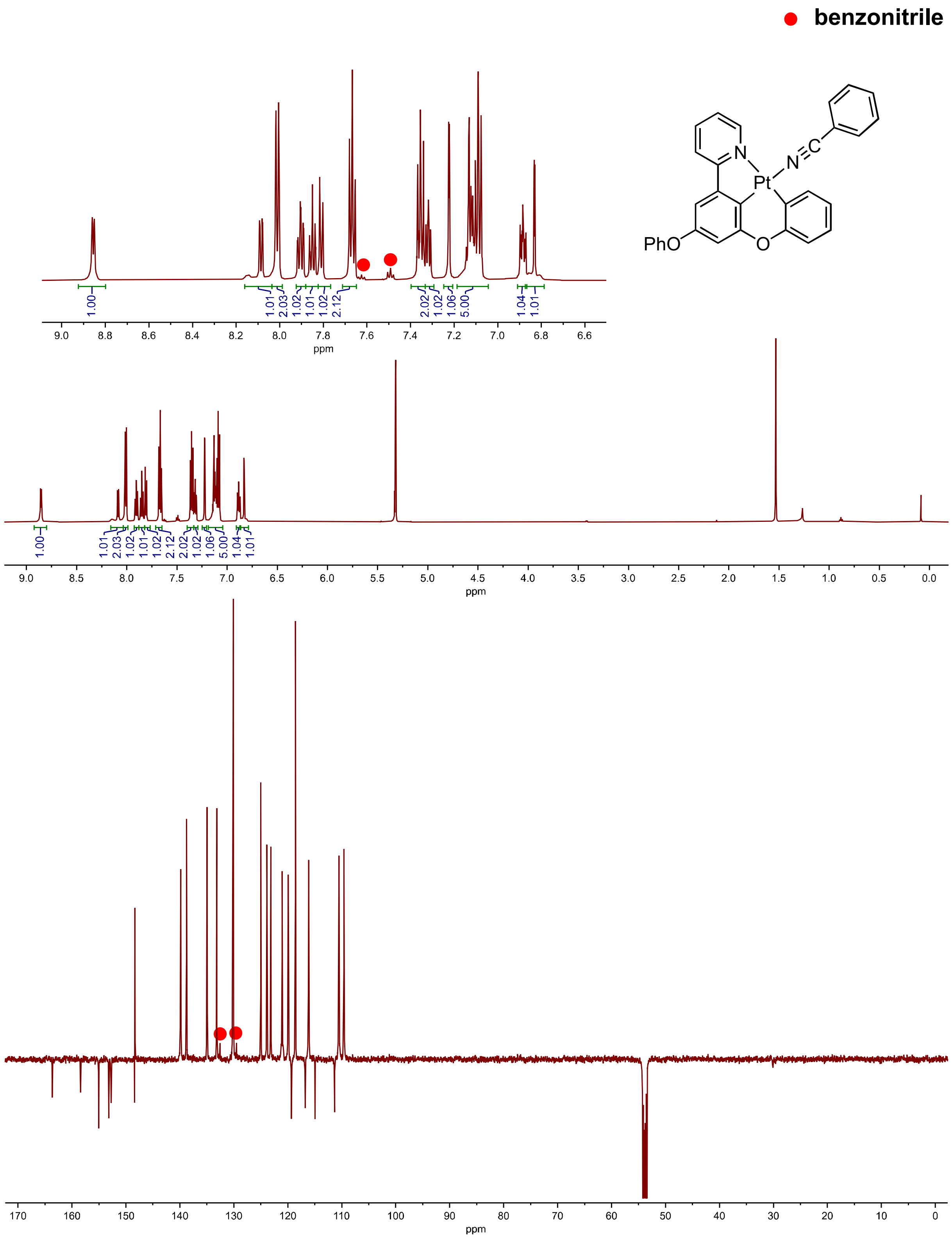


**Figure S15.** $^{1}H$ (top) and $^{13}C\{^{1}H\}$ APT (bottom) NMR spectra of complex **10** ($CD_2Cl_2$, 600 and 151 MHz, respectively).

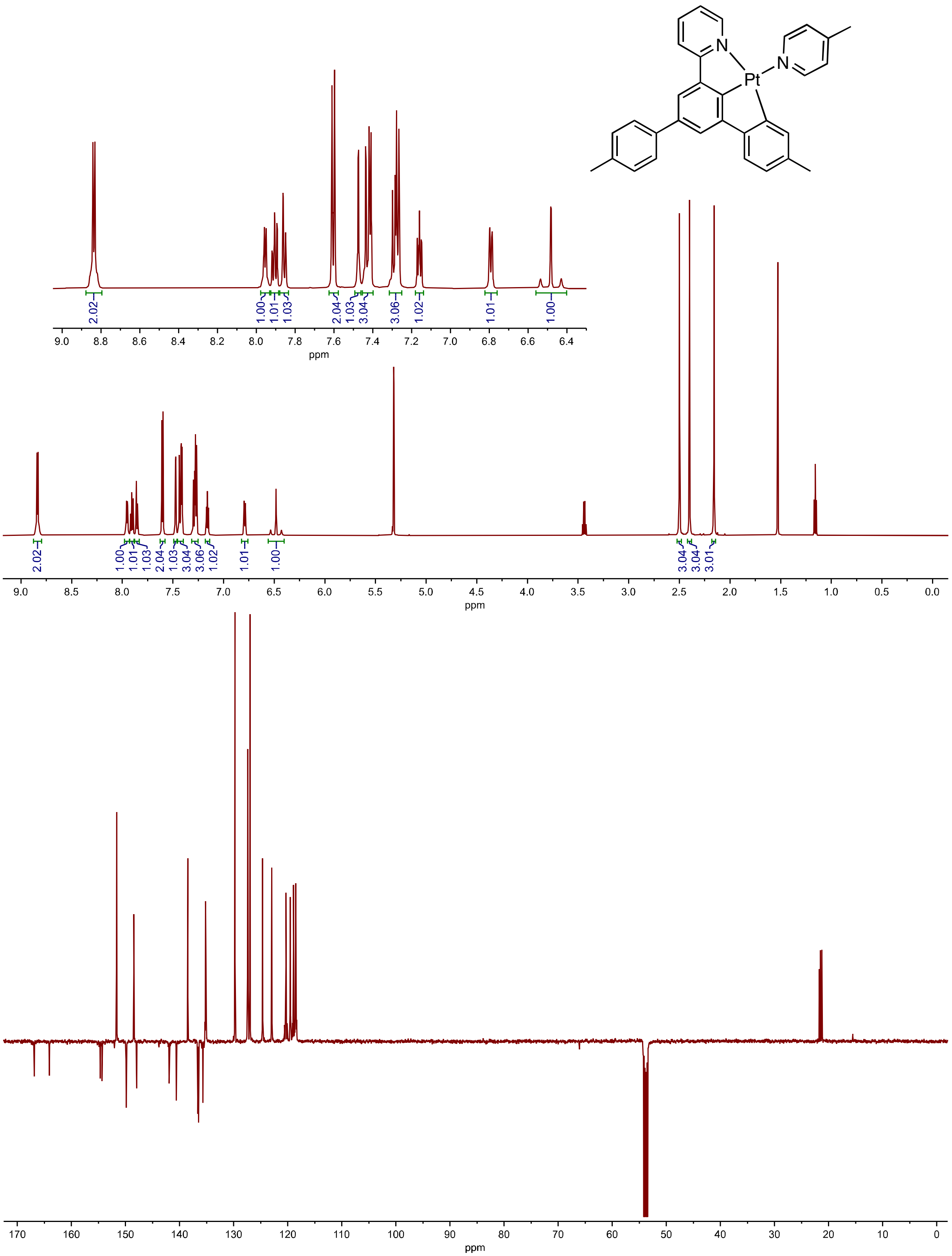


**Figure S16.** $^{1}H$ (top) and $^{13}C\{^{1}H\}$ APT (bottom) NMR spectra of complex **11** ($CD_2Cl_2$, 600 and 151 MHz, respectively).

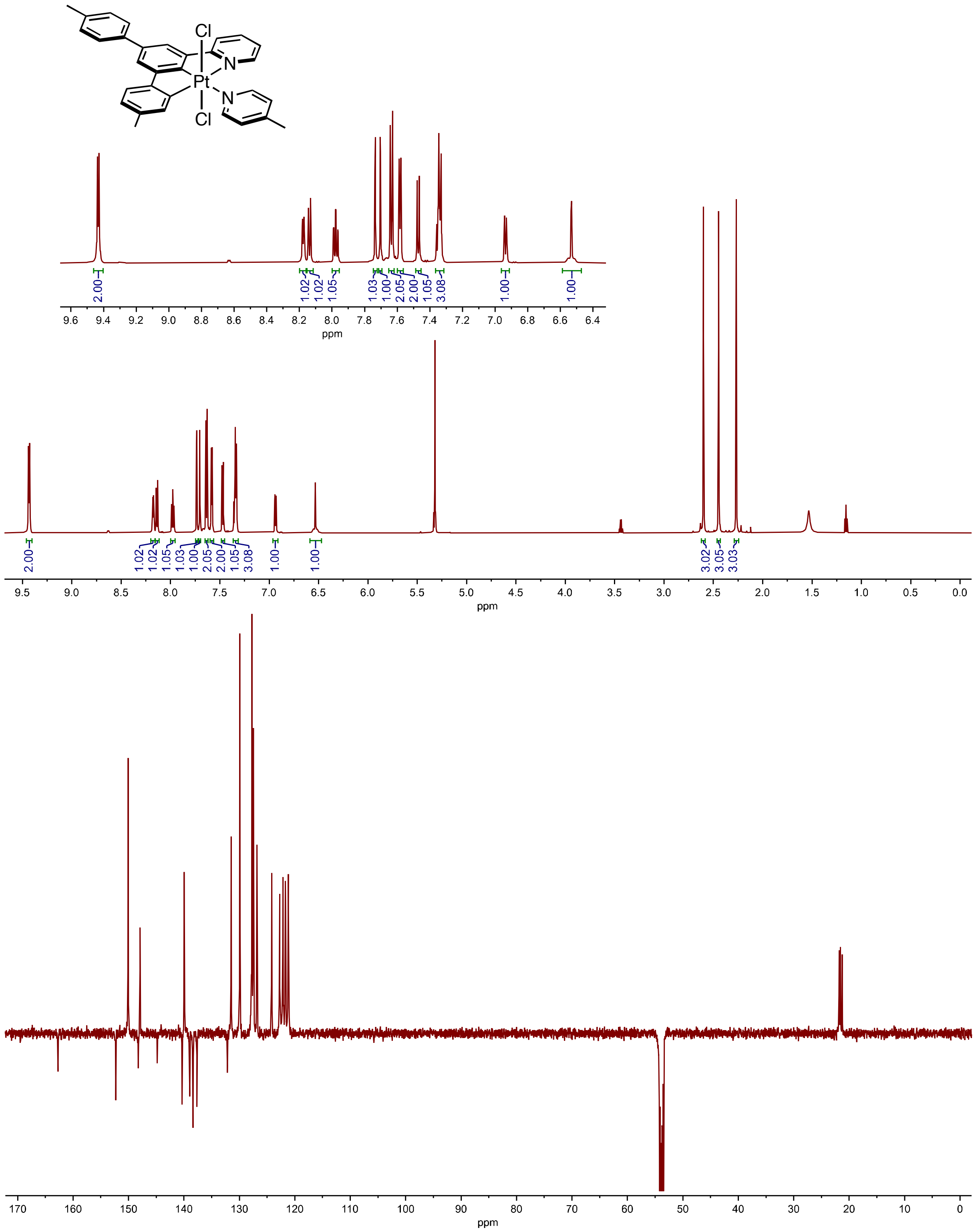


**Figure S17.** $^{1}H$ (top) and $^{13}C\{^{1}H\}$ APT (bottom) NMR spectra of complex **13** ($CD_2Cl_2$, 600 and 151 MHz, respectively).

# 3. Reaction monitoring by $^1$H NMR

## 3.1. Photochemical generation of $Bu_4N[PtCl_2(ppy)]$ from $(Bu_4N)_2[Pt_2Cl_6]$ and ppyH

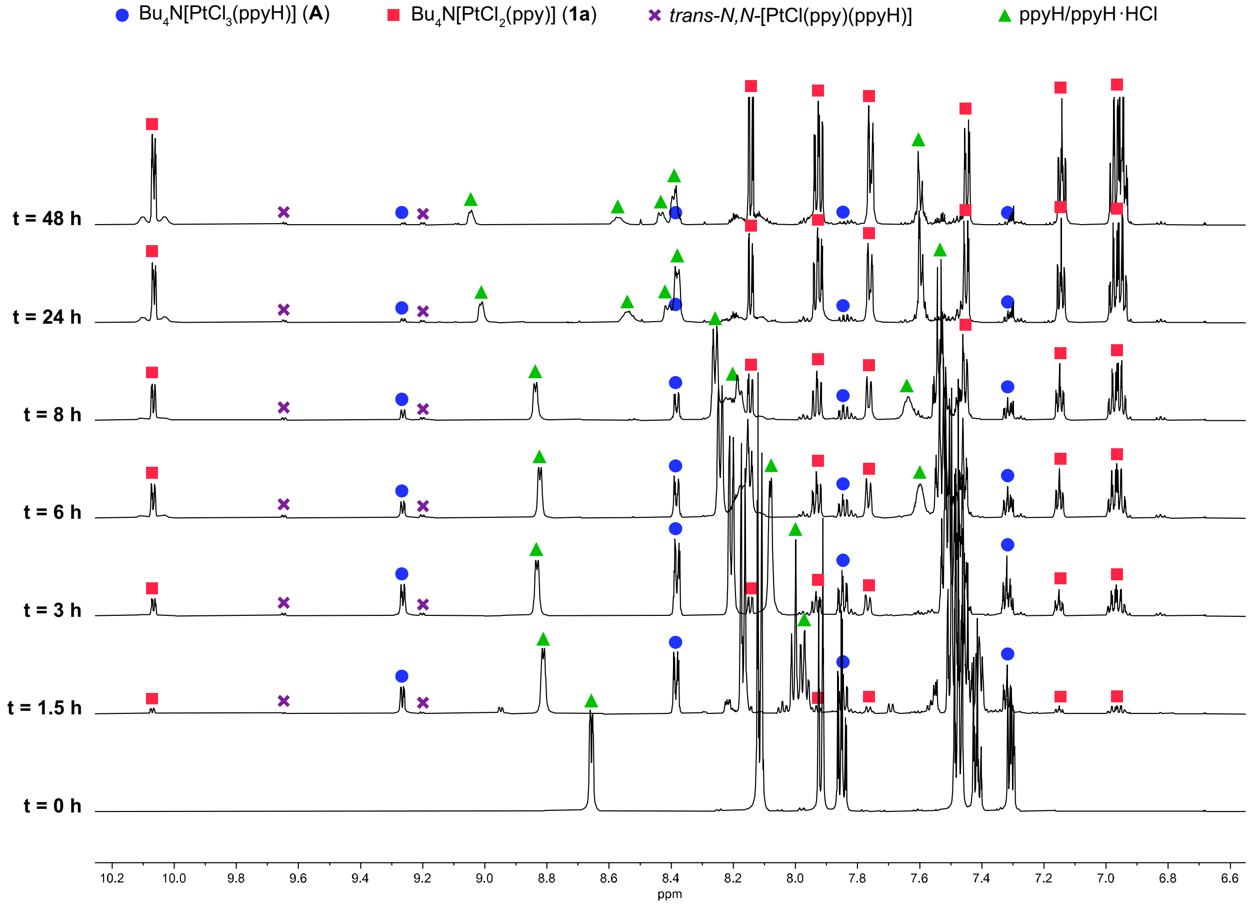


**Figure S18.** $^1$H NMR spectra (600 MHz) of an acetone-$d_6$ solution of $(Bu_4N)_2[Pt_2Cl_6]$, ppyH and piperidinomethyl(polystyrene) after different times of irradiation with green LEDs at room temperature (aromatic region). Conditions: $(Bu_4N)_2[Pt_2Cl_6]$ (10 mg, 0.01 mmol), ppyH (3 µL, 0.02 mmol), piperidinomethyl(polystyrene) (11 mg, *ca.* 0.04 mmol), acetone-$d_6$ (0.5 mL). Note that the base is not totally effective in capturing the released HCl and part of the free ppyH is protonated to give ppyH·HCl, resulting in a gradual deshielding of the resonance of the H ortho to the N atom.

**Table S5.** Product yields (%) with respect to $(Bu_4N)_2[Pt_2Cl_6]$.[a]

| Irradiation time (h) | $Bu_4N[PtCl_3(ppyH)]$ (A) | 1a | *trans-N,N*-[PtCl(ppy)(ppyH)] |
|---|---|---|---|
| 0 | 0 | 0 | 0 |
| 1.5 | 14 | 3 | 1 |
| 3 | 18 | 11 | 1 |
| 6 | 9 | 21 | 2 |
| 8 | 6 | 26 | 2 |
| 24 | 3 | 47 | 2 |
| 48 | 1 | 60 | 2 |

[a]Measured using the $CH_2$ protons of the $Bu_4N^+$ cation as standard.

**Identification of intermediate A.** Only four resonances corresponding to the coordinated ppyH ligand of intermediate **A** can be clearly distinguished in the $^{1}$H NMR spectra of the monitoring experiment (Figure S18). Two of them can be unequivocally assigned on the basis of the $^{1}$H NMR data of *trans-N,N*-[PtCl(ppy)(ppyH)].[26,27] The resonance at 9.27 ppm is somewhat broadened at the base because of unresolved Pt satellites and corresponds to the proton ortho to the N atom of the coordinated ppyH. The resonance at 8.38 ppm that integrates as 2 protons with respect to the previous one can be assigned to the protons in positions 2 and 6 of the pendant phenyl ring, proving that the ppyH ligand has not undergone cyclometalation. Therefore, this intermediate can only be the anionic complex [PtCl$_3$(ppyH)]$^{-}$.

## 3.2. Stability of [PtCl$_3$(ppyH)]$^{-}$ in the dark

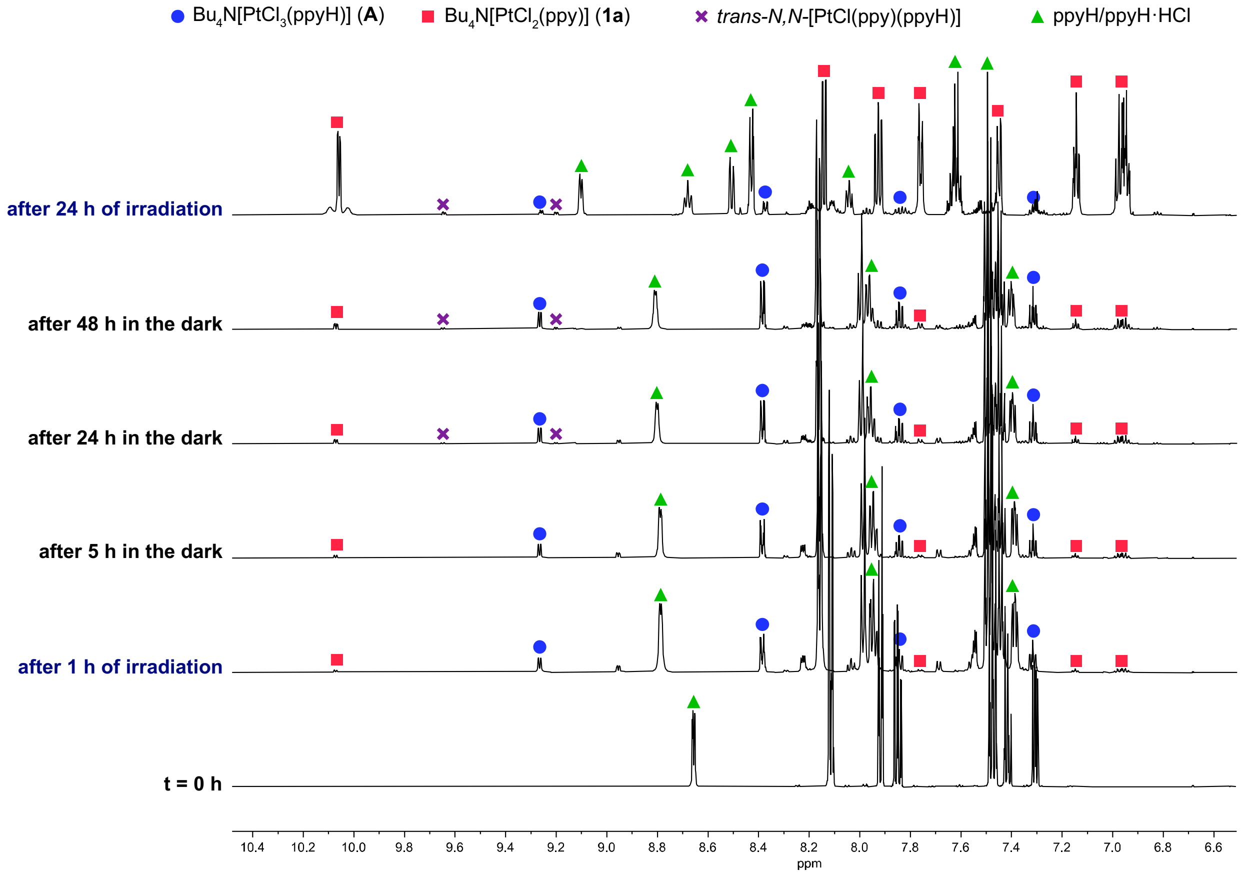


**Figure S19.** $^{1}$H NMR spectra (600 MHz) of an acetone-$d_6$ solution of $(Bu_4N)_2[Pt_2Cl_6]$, ppyH and piperidinomethyl(polystyrene) after different times under irradiation with green LEDs or in the dark at room temperature (aromatic region). Conditions: $(Bu_4N)_2[Pt_2Cl_6]$ (10 mg, 0.01 mmol), ppyH (3 µL, 0.02 mmol), piperidinomethyl(polystyrene) (11 mg, *ca.* 0.04 mmol), acetone-$d_6$ (0.5 mL).

**Table S6.** Product yields (%) with respect to $(Bu_4N)_2[Pt_2Cl_6]$.[a]

| **Spectrum** | **$(Bu_4N)[PtCl_3(ppyH)]$** | **1a** | ***trans-N,N*-[PtCl(ppy)(ppyH)]** |
|---|---|---|---|
| t = 0 h | 0 | 0 | 0 |
| After 1 h of irradiation | 9 | 1 | 0 |
| After 5 h in the dark | 10 | 2 | 0 |
| After 24 h in the dark | 11 | 3 | 1 |
| After 48 h in the dark | 12 | 4 | 2 |
| After 24 h of irradiation | 2 | 54 | 2 |

[a]Measured using the $CH_2$ protons of the $Bu_4N^{+}$ cation as standard.

## 3.3. Photochemical reaction between $[PtCl_2(NCPh)_2]$ and tpyH

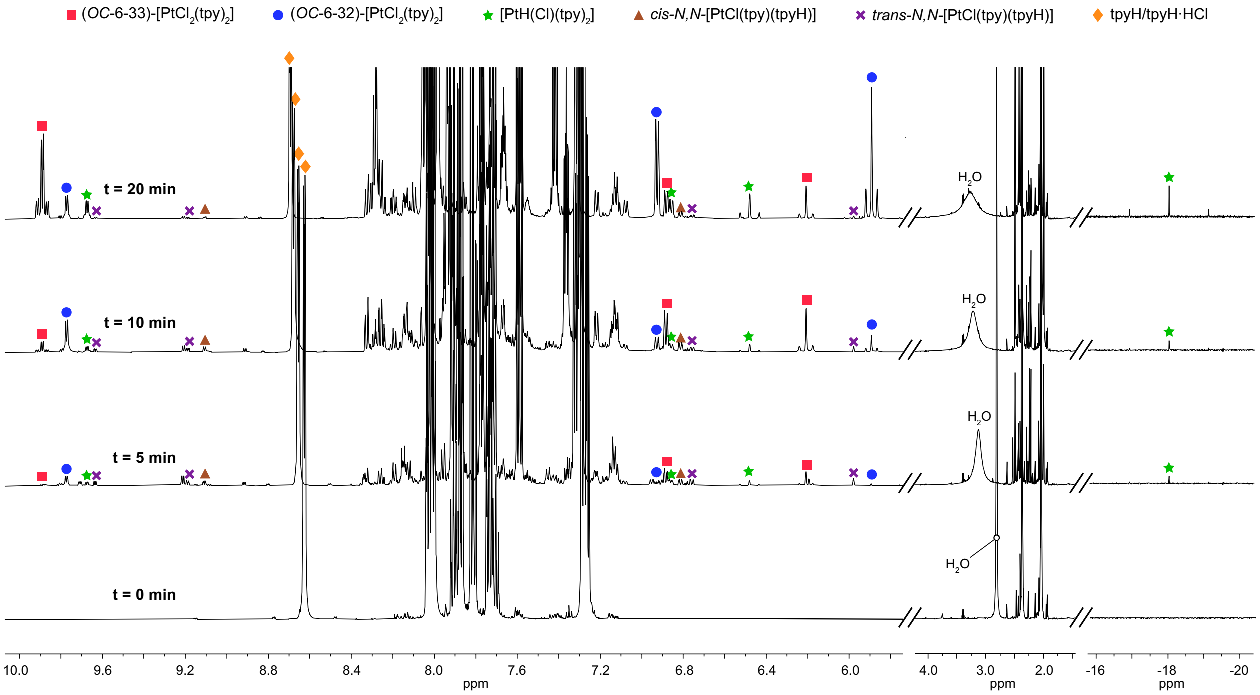


**Figure S20.** $^{1}$H NMR spectra (600 MHz) of an acetone-$d_6$ solution of $[PtCl_2(NCPh)_2]$ and tpyH after different times under irradiation with a violet LED (aromatic, aliphatic and hydride regions). Conditions: $[PtCl_2(NCPh)_2]$ (5 mg, 0.01 mmol), tpyH (5 µL, 0.03 mmol), acetone-$d_6$ (0.5 mL).

**Additional comments.** In this experiment, complex (*OC*-6-32)-$[PtCl_2(tpy)_2]$[28] (Scheme S2) presumably arises from the reaction between $[PtH(Cl)(tpy)_2]$ and HCl released from the electrophilic metalation of the first tpyH ligand. However, the major product after 20 min of irradiation is its $C_2$-symmetrical isomer (*OC*-6-33)-$[PtCl_2(tpy)_2]$, which must be produced upon photoisomerization.[29]

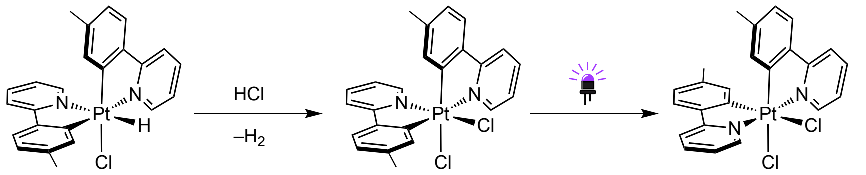


$[PtH(Cl)(tpy)_2]$ (*OC*-6-32)-$[PtCl_2(tpy)_2]$ (*OC*-6-33)-$[PtCl_2(tpy)_2]$

**Scheme S2.**

# 4. References


(1) Coulson, D. R.; Satek, L. C.; Grim, S. O. Tetrakis(Triphenylphosphine)Palladium(0). *Inorg. Synth.* **1972**, *13*, 121–124.

(2) Uchiyama, T.; Toshiyasu, Y.; Nakamura, Y.; Miwa, T.; Kawaguchi, S. The Isolation, Characterization, and Isomerization of Cis- and Trans-Bis(Benzonitrile)Dichloroplatinum(II). *Bull. Chem. Soc. Jpn.* **1981**, *54*, 181–185.

(3) Kong, P.-C.; Rochon, F. D. Reactions of K2PtCl4 with Pyridine Derivatives in Dimethylformamide and Synthesis of Potassium Trichloro(Pyridine)Platinum(II). *Can. J. Chem.* **1978**, *56*, 441–445.

(4) Charles, R. G. Tetraacetylethane. *Org. Synth.* **1959**, *39*, 61.

(5) Yang, X.; Sun, N.; Dang, J.; Huang, Z.; Yao, C.; Xu, X.; Ho, C.-L.; Zhou, G.; Ma, D.; Zhao, X.; Wong, W.-Y. Versatile Phosphorescent Color Tuning of Highly Efficient Borylated Iridium(III) Cyclometalates by Manipulating the Electron-Accepting Capacity of the Dimesitylboron Group. *J. Mater. Chem. C* **2013**, *1*, 3317–3326.

(6) Tsuboyama, A.; Iwawaki, H.; Furugori, M.; Mukaide, T.; Kamatani, J.; Igawa, S.; Moriyama, T.; Miura, S.; Takiguchi, T.; Okada, S.; Hoshino, M.; Ueno, K. Homoleptic Cyclometalated Iridium Complexes with Highly Efficient Red Phosphorescence and Application to Organic Light-Emitting Diode. *J. Am. Chem. Soc.* **2003**, *125*, 12971–12979.

(7) Constable, E. C.; Henney, R. P. G.; Leese, T. A.; Tocher, D. A. Cyclometallation Reactions of 6-Phenyl-2,2′-Bipyridine; a Potential C,N,N-Donor Analogue of 2,2′:6′,2″-Terpyridine. Crystal and Molecular Structure of Dichlorobis(6-Phenyl-2,2′-Bipyridine)Ruthenium(II). *J. Chem. Soc., Dalton Trans.* **1990**, 443–449.

(8) Cárdenas, D. J.; Echavarren, A. M.; Ramírez De Arellano, M. C. Divergent Behavior of Palladium(II) and Platinum(II) in the Metalation of 1,3-Di(2-Pyridyl)Benzene. *Organometallics* **1999**, *18*, 3337–3341.

(9) Wang, Z.; Turner, E.; Mahoney, V.; Madakuni, S.; Groy, T.; Li, J. Facile Synthesis and Characterization of Phosphorescent Pt(N^C^N)X Complexes. *Inorg. Chem.* **2010**, *49*, 11276–11286.

(10) Li, Q.; Shi, C.; Zhang, X.; Tao, P.; Zhao, Q.; Yuan, A. Comparison of Structural and Optical Properties for N-Embedded Polycyclic and Non-Embedded Cationic Phosphorescent Iridium(III) Complexes. *Eur. J. Inorg. Chem.* **2019**, 1343–1348.

(11) Juliá, F.; González-Herrero, P. Aromatic C-H Activation in the Triplet Excited State of Cyclometalated Platinum(II) Complexes Using Visible Light. *J. Am. Chem. Soc.* **2016**, *138*, 5276–5282.

(12) Goggin, P. L. Stretching Vibrations of Planar Di-μ-Halogeno-Bisdihalogeno-Palladate(II) and -Platinate(II) Anions. *J. Chem. Soc., Dalton Trans.* **1974**, 1483–1486.

(13) Kumar, R.; Linden, A.; Nevado, C. Luminescent (N^C^C) Gold(III) Complexes: Stabilized Gold(III) Fluorides. *Angew. Chem. Int. Ed.* **2015**, *54*, 14287–14290.

(14) Craig, C. A.; Garces, F. O.; Watts, R. J.; Palmans, R.; Frank, A. J. Luminescence Properties of Two New Pt(II)-2-Phenylpyridine Complexes; the Influence of Metal-Carbon Bonds. *Coord. Chem. Rev.* **1990**, *97*, 193–208.

(15) Aoki, R.; Kobayashi, A.; Chang, H. C.; Kato, M. Structures and Luminescence Properties of Cyclometalated Dinuclear Platinum(II) Complexes Bridged by Pyridinethiolate Ions. *Bull. Chem. Soc. Jpn.* **2011**, *84*, 218–225.

(16) Martínez-Junquera, M.; Lalinde, E.; Moreno, M. T. Multistimuli-Responsive Properties of Aggregated Isocyanide Cycloplatinated(II) Complexes. *Inorg. Chem.* **2022**, *61*, 10898–10914.

(17) Kvam, P.-I.; Songstad, J.; Hanson, J. C.; Songstad, J.; Lundberg, C.; Arnarp, J.; Björk, L.; Gawinecki, R. Preparation and Characterization of Some Cyclometalated Pt(II) Complexes from 2-Phenylpyridine and 2-(2’-Thienyl)Pyridine. *Acta Chem. Scand.* **1995**, *49*, 313–324.

(18) Hudson, Z. M.; Blight, B. A.; Wang, S. Efficient and High Yield One-Pot Synthesis of Cyclometalated Platinum(II) β-Diketonates at Ambient Temperature. *Org. Lett.* **2012**, *14*, 1700–1703.

(19) Brooks, J.; Babayan, Y.; Lamansky, S.; Djurovich, P. I.; Tsyba, I.; Bau, R.; Thompson, M. E. Synthesis and Characterization of Phosphorescent Cyclometalated Platinum Complexes. *Inorg. Chem.* **2002**, *41*, 3055–3066.

(20) Chassot, L.; von Zelewsky, A. Cyclometalated Complexes of Platinum(II): Homoleptic Compounds with Aromatic C,N Ligands. *Inorg. Chem.* **1987**, *26*, 2814–2818.

(21) López-López, J. C.; Bautista, D.; González-Herrero, P. Stereoselective Formation of Facial Tris-Cyclometalated Pt(IV) Complexes: Dual Phosphorescence from Heteroleptic Derivatives. *Chem. Eur. J.* **2020**, *26*, 11307–11315.

(22) Cheung, T.; Che, C. Photoluminescent Cyclometallated Diplatinum(II,II) Complexes: Photophysical Properties and Crystal Structures of [PtL(PPh3)]ClO4 and [Pt2L2(μ-Dppm)][ClO4]2 (HL = 6-Phenyl-2,2′-

Bipyridine, Dppm = Ph2PCH2PPh2). *J. Chem. Soc., Dalton Trans.* **1996**, 1645–1651.
(23) Williams, J. A. G.; Beeby, A.; Davies, E. S.; Weinstein, J. A.; Wilson, C. An Alternative Route to Highly Luminescent Platinum(II) Complexes:Cyclometalation with N^C^N-Coordinating Dipyridylbenzene Ligands. *Inorg. Chem.* **2003**, *42*, 8609–8611.
(24) Sheldrick, G. M. A Short History of SHELX. *Acta Crystallogr., Sect. A Found. Crystallogr.* **2008**, *64*, 112–122.
(25) Sheldrick, G. M. SHELXT – Integrated Space-Group and Crystal-Structure Determination. *Acta Crystallogr. Sect. A Found. Adv.* **2015**, *71*, 3–8.
(26) Niedermair, F.; Waich, K.; Kappaun, S.; Mayr, T.; Trimmel, G.; Mereiter, K.; Slugovc, C. Heteroleptic 2-Phenylpyridine Platinum Complexes: The Use of Bis(Pyrazolyl)Borates as Ancillary Ligands. *Inorg. Chim. Acta* **2007**, *360*, 2767–2777.
(27) Fukuda, H.; Yamada, Y.; Hashizume, D.; Takayama, T.; Watabe, M. [Pt(Topy)(Htopy)(ONO2)] Complex (Htopy = 2-p-Tolylpyridine) and Its Analogs: 195Pt NMR Spectra and Fabrication of Light-Emitting Devices. *Appl. Organomet. Chem.* **2009**, *23*, 154–160.
(28) Vivancos, Á.; Poveda, D.; Muñoz, A.; Moreno, J.; Bautista, D.; González-Herrero, P. Selective Synthesis, Reactivity and Luminescence of Unsymmetrical Bis-Cyclometalated Pt(IV) Complexes. *Dalton Trans.* **2019**, *48*, 14367–14382.
(29) Juliá, F.; García-Legaz, M.-D.; Bautista, D.; González-Herrero, P. Influence of Ancillary Ligands and Isomerism on the Luminescence of Bis-Cyclometalated Platinum(IV) Complexes. *Inorg. Chem.* **2016**, *55*, 7647–7660.
